# Cotton field population phenotyping analysis based on 3D Gaussian reconstruction and dynamic spatial constraints

**DOI:** 10.3389/fpls.2026.1790984

**Published:** 2026-04-10

**Authors:** Bo Liu, Xiaojuan Li, Jiajie Yang, Zhi Liang, Xiaotong Zheng, Xiangjun Zou

**Affiliations:** 1 School of Mechanical Engineering, Xinjiang University, Urumqi, China; 2 State Key Laboratory of Cotton Bio-breeding and Integrated Utilization, Institute of Cotton Research, Chinese Academy of Agricultural Sciences, Anyang, China

**Keywords:** cotton, deep learning, plant phenotyping, point cloud segmentation, semantic segmentation, three-dimensional reconstruction

## Abstract

**Introduction:**

High-throughput field phenotyping (HTFP) holds great potential for elucidating the relationship between genomes and phenotypes. However, obtaining high-quality three-dimensional point cloud data of field populations and achieving single-plant phenotypic analysis remain challenging.

**Methods:**

This study develops an integrated framework for field crop reconstruction based on 3D Gaussian splatting, incorporating a geometry-aware dynamic constraint algorithm to achieve instance segmentation and extract key phenotypic traits of individual plants. Using 3D Gaussian splatting technology, field-scale cotton population modeling is accomplished, generating dense 3D point clouds for regions of interest. Furthermore, the concept of a crop localization domain is proposed, establishing a longitudinal mapping that associates plant positional coordinates with long-term phenotypic attributes. Finally, through a dynamic spatial constraint mechanism, the accuracy and computational efficiency of instance segmentation for crop population point clouds are significantly improved, enabling rapid extraction of individual plant traits such as cotyledon node height, plant height, and leaf area.

**Results:**

The results demonstrate that PhenotypeAI successfully reconstructed nine cotton populations with PSNR exceeding 30.0 dB. It successfully extracted regions of interest from 403 cotton plants, achieving an average F-score of 91.32% for instance segmentation and an average accuracy of 91.35%. The extracted traits—cotyledon node height, plant height, and leaf area—exhibited strong correlations with manual measurements, with coefficients of determination (*R*^2^) of 0.90, 0.91, and 0.91, respectively.

**Discussion:**

The proposed method provides a low-cost solution for high-throughput field phenotypic analysis of field cotton and improves the efficiency of cotton breeding.

## 1 Introduction

Cotton, as one of the world’s most important agricultural products, is the most widely cultivated natural fiber crop globally (Jiang et al., 2025; Yu et al., 2025). It finds extensive applications in textiles, food, medicine, construction, and other sectors, holding significant importance in international trade (Li et al., 2020). Faced with challenges such as climate change and resource depletion, there is an urgent need to develop high-quality cotton varieties to promote the high-quality development of the cotton industry (Abdurakhimov et al., 2023). Cotton breeding technology requires the evaluation of multiple traits across thousands of breeding lines through multi-year, multi-location trials with multiple replicates, screening high-quality varieties from vast sample populations (Chawade et al., 2019; Dipta et al., 2023). Consequently, the rapid and accurate acquisition and analysis of plant phenotypic information constitute a critical component of variety selection and breeding (He et al., 2024). However, cotton exhibits a vast array of varieties, rich phenotypic information, and complex biological characteristics (Chen et al., 2024; Iqbal et al., 2020). The acquisition of its phenotypic information has long remained confined to traditional manual methods, characterized by low throughput, inconsistent standards, and typically destructive, labor-intensive, and time-consuming procedures (Bian et al., 2022), making it challenging to accurately obtain phenotypic data for individual crops at the population scale (Fu and Jiang, 2022). Furthermore, manual measurement of phenotypic parameters typically allows sampling only at fixed time points, failing to provide continuous monitoring of the dynamic developmental processes and variation characteristics within the same plant. There is an urgent need for automated, high-precision, and low-cost technologies for phenotyping acquisition and analysis in field trial plots.

Visual-based target and crop identification and detection methods, primarily encompassing deep learning (Tang et al., 2020), point cloud reconstruction (Li et al., 2024; Peng et al., 2025), and color segmentation techniques (Liang et al., 2025), have achieved significant progress. Notably, the integration of 3D point cloud reconstruction with plant analysis technology is emerging as an automated alternative for capturing phenotypic information. While LiDAR scanning (Chen et al., 2025; Gu et al., 2024; Ma et al., 2024) can precisely capture crop phenotypic parameters, its low efficiency, high cost, and lack of fine-grained details in reconstructed plant point clouds limit its applicability for phenotypic measurements. Structure from Motion (SFM) and Multi-View Stereo (MVS) estimate scene structure by triangulating keypoints across multiple images. These methods have been widely applied in extracting phenotypic parameters for crops such as soybean (Cui et al., 2025; He et al., 2023), sugar beet (Chen et al., 2024), and maize (Gao et al., 2023). However, due to limitations in feature matching and dense reconstruction algorithms, this approach often fails to generate complete and detailed point clouds. Neural Radiation Fields (NeRF) (Tancik et al., 2023) and other implicit neural representation techniques demonstrate significant potential in overcoming the limitations of traditional Structure from Motion-Model-Based Visualization (SfM-MVS) methods. They can reconstruct high-quality 3D models with superior performance in occluded regions and plant detail. When applied to the three-dimensional reconstruction of field populations, conventional methods based on Structure-from-Motion (SFM) and Multi-View Stereo (MVS) using RGB imagery tend to generate considerable noise along leaf margins and produce fractured point clouds for plant stems (Gu et al., 2024). NeRF-based methods effectively reduce edge noise and mitigate point cloud fragmentation by leveraging neural networks to learn scene geometry and appearance. However, this approach requires intensive sampling of the neural network to generate point clouds, making it more suitable for small-scale studies or simple trait measurements on limited plant populations (F. Saeed et al., 2023). Three-dimensional Gaussian Splatter (3DGS) (Bao et al., 2025; Kerbl et al., 2023), a point-based 3D representation technique, constructs spatially distributed Gaussian primitives based on observed viewpoints. This generates explicit expressions of scene color distribution and texture features, enabling real-time rendering while maintaining high visual quality. It has emerged as a more efficient 3D reconstruction solution. Currently, with breakthrough advancements in algorithms such as Neural Radiance Fields (NeRF) and 3D Gaussian Splatting, multi-view imaging using smartphones has demonstrated significant potential for 3D reconstruction at the organ level in plants. This enables high-quality 3D reconstruction of plant populations in field conditions, offering substantial advantages in terms of low cost and convenience.

Obtaining single plant crops from field populations through point cloud instance segmentation is a crucial prerequisite for automatic, accurate, and non-invasive acquisition of plant phenotypic information (Digumarti et al., 2018; Elnashef et al., 2019; Wang et al., 2025a). Currently, the main methods for instance segmentation of point clouds in crop populations include those based on classical algorithms (Mirande et al., 2022; Zou et al., 2023) and those based on deep learning networks (Dong et al., 2025; J and Nidamanuri, 2024; Li et al., 2023; Wang et al., 2025b). Mirande et al. (2022) achieved plant point cloud segmentation and extracted target phenotypic parameters based on agronomic knowledge. Chen et al. (2025) employed the PointNet++ method to achieve semantic segmentation of LiDAR point clouds in cotton fields, successfully extracting cotton point clouds at both the single-plant and plot scales. Wang et al. (2025b) proposed the rubber tree point cloud segmentation network RsegNet, achieving a best F-score of 86.1% for rubber tree segmentation on the FOR-instance forest dataset. Deep learning has demonstrated impressive performance in plant point cloud instance segmentation, but its training relies on massive, high-precision annotated datasets. Zhang et al. (2025) proposed an innovative method named Wheat3DGS, which integrates 3D Gaussian splatting (3DGS) with the Segment Anything Model (SAM), to achieve field point cloud reconstruction of wheat canopy parts, instance segmentation of wheat spike organs, and phenotypic measurement. The method uses a pre-trained wheat spike detector (YOLOv5) to generate SAM prompts for obtaining 2D segmentation masks, projects these masks into the 3DGS-reconstructed scene, and achieves multi-view mask association and 3D instance segmentation through an iterative matching fine-tuning strategy. This method targets the wheat canopy spike parts, which are characterized by relatively uniform morphology, dense distribution, and compactness. In contrast, field cotton populations present challenges due to inter-plant branch entanglement, canopy adhesion, and significant variation in appearance features such as leaf orientation and branch morphology, lacking stable and consistent appearance characteristics. This determines that the strategy of transferring YOLO detectors and 2D mask projection, which relies on appearance priors, is difficult to apply effectively to cotton. Achieving precise semantic segmentation of complete cotton plants in high-density, compact cotton field environments remains a challenging task. Existing methods have not fully addressed the challenges frequently encountered when segmenting individual cotton plants, especially in high-density and compact cotton field environments. The main issues are as follows: (1) In high-density cotton populations, inter-plant branch entanglement, canopy adhesion, and variable leaf orientation and occlusion states result in a lack of stable, distinguishable appearance features. This complexity makes it difficult for appearance-based deep learning models to learn robust feature representations. More critically, performing pixel-level or instance-level manual precise annotation for complete field cotton population point clouds is difficult, costly, and error-prone, limiting the feasibility of supervised learning frameworks. (2) As a densely planted, low-growing crop, cotton plants within a population exhibit high similarity in three-dimensional geometric morphology, while also having uneven planting spacing and potential random offsets between plants. This makes traditional unsupervised methods that rely solely on local point cloud density or general geometric features highly prone to over-segmentation or under-segmentation at plant boundaries. (3) Despite macroscopic similarity among plants in the population, individual plants still exhibit differences in structural parameters such as height, canopy width, and branching angle. Segmentation methods that ignore the vertical distribution of crops and the topological properties of individual plant morphology struggle to accurately define plant boundaries, reducing segmentation accuracy and reliability. This paper focuses on the inherent, stable prior knowledge and topological properties of the geometric structure of cotton plants, and implements a training-free, annotation-free instance segmentation method.

In summary, this study develops an integrated framework for field crop reconstruction based on 3D Gaussian Splatting, incorporating a geometry-aware dynamic constraint algorithm to achieve instance segmentation. This framework can accurately capture the three-dimensional morphological structure of individual cotton plants, which is of significant importance for the precise measurement of cotton phenotypic parameters and breeding work. The main contributions are as follows:

1. A high-quality 3D point cloud reconstruction pipeline for field cotton populations is proposed. Based on 3D Gaussian Splatting (3DGS) for cotton population reconstruction, point cloud data is sampled via Gaussian distribution probability, and outliers are eliminated using a Mahalanobis distance threshold, ultimately generating high-density, high-precision point clouds of cotton populations.
2. We introduced the concept of a plant localization zone. By generating non-convex clusters from the dataset using the Density-Based Spatial Clustering of Applications with Noise (DBSCAN) algorithm, we determined the location of each cotton plant and established a longitudinal mapping relationship between plant position information and phenotypic data.
3. We incorporated the vertical distribution and morphological structure of cotton plants within a group into the constraint scope through a dynamic spatial constraint approach. This enabled the establishment of an improved regional growth method for segmenting cotton plant groups, allowing the group point cloud to rapidly segment plant point clouds within a predetermined constrained space while adapting to variations in the size of the cotton group.
4. To validate the method’s effectiveness, extensive experiments were conducted. Quantitative and qualitative analyses were performed on field-collected cotton group datasets to evaluate reconstruction and segmentation performance. Point cloud data from 403 multi-varietal cotton plants were successfully acquired. Based on segmentation results, key structural parameters of individual plants—including cotyledon node height, plant height, leaf count, and leaf area—were successfully measured.

## 2 Materials and methods

### 2.1 Field trial design and cotton plant material

**Figure 1:**
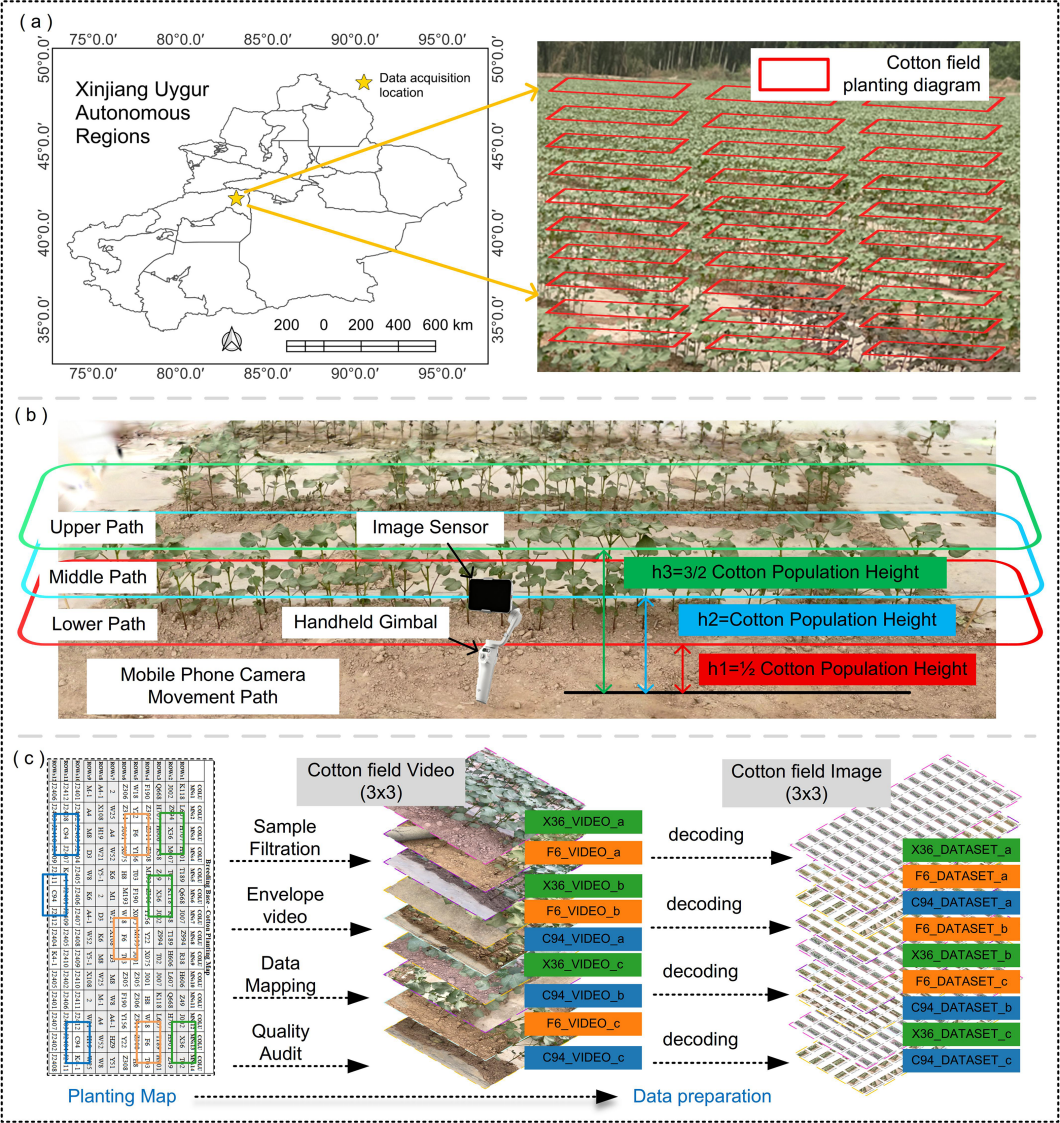
Experimental fields and data acquisition. **(a)** Experiment location. **(b)** Sensor height and motion path during data acquisition. **(c)** Locate sample plots based on the planting map, acquire the sample plot envelope imagery data, and extract key frames.

The experiment was conducted at the Kuqa Modern Agricultural Breeding Center in China (longitude: 82*^°^* 97', latitude: 41° 68'). The breeding field implemented lean agronomic management, including regular chemical weeding and manual inspection, resulting in only sporadic and minor weeds in the field. Prior to planting, land leveling and soil preparation were carried out, and plastic film mulch was laid, making the field surface relatively flat. The planting pattern is shown in **Figure 1**, featuring plot cultivation of different varieties. Breeding experts selected three new varieties suitable for mechanical harvesting from the breeding field: ‘Kechuang 94’, ‘Fuzimian 6’, and ‘Xinluzao 36’. Each variety was arranged in three replicate plots, totaling nine plots. Each sample plot measured 2.0 m × 2.0 m. Precision seeding was performed using a precision seeder with uniform spacing: row spacing of 660 mm (25.98 inches) and plant spacing of 100 mm (3.94 inches), with one plant per hill. The effective plant counts were as follows: VARc94 (133 plants), VARf6 (132 plants), VARx36 (138 plants), with a total population size of 403 plants.

The collected data pertains to the 6–7 leaf stage of cotton plants. Key phenotypic traits of focus during this period include plant height, cotyledon node height, number of leaves, and leaf area. Field data collection must be conducted under stable lighting conditions with wind speeds *≤* 3* m/s* on the Beaufort scale. Avoid extreme weather and significant crop sway to ensure clear image textures and accurate point cloud registration. Operations should be conducted between 13:00 and 15:00. The experiment employs a stratified random sampling design: based on the spatial distribution map of the breeding field, nine independent sample plots are allocated to the three varieties VARc94, VARx36, and VARf6 in a checkerboard pattern. A minimum isolation zone of *≥* 2* m* between plots is maintained to eliminate edge effects. The data acquisition system is built using an iPhone 12 smartphone terminal, with specific parameters detailed in **Table 1**. The operator moves clockwise along the sample area at a uniform pace, maintaining an average distance of approximately 0.8 meters between the acquisition terminal and the target cotton plants. Continuous 2K video recording is conducted for 120 seconds. Video data underwent frame parsing using FFmpeg 5.0, employing an equidistant sampling strategy to extract 840 frames per plot. Lens distortion correction and automatic white balance processing were performed via OpenCV 4.5, ultimately generating a 7,560 frame multi-perspective image dataset.

**Table 1:** Key parameters of data acquisition platform.

Parameters	Value
Sensor diagonal (mm)	7.62
Focal length (mm)	26
Image width (pixels)	1920
Image height (pixels)	1080
Angle of view (°)	120

Phenotypic Measurement Data Collection: The ST-500 stainless steel ruler was used to measure the plant height of cotton vertically from the cotyledon node of the main stem to the top growth point of the plant. The steel ruler was kept parallel to the axis of the main stem during the measurement. Each plant was measured three times repeatedly, and the arithmetic mean value was taken as the final value. Align the zero point of the tape measure with the stem base and follow the main stem’s curvature to measure the height to the first leaf node. Repeat this measurement three times, taking the arithmetic mean as the final value. Perform non-destructive leaf area measurements using the YMJ-D handheld live leaf area meter. Select 30 functional leaves per sample plot using systematic sampling, with 10 leaves each from the upper, middle, and lower canopy layers. To achieve precise matching of predicted leaves with manually measured samples, we utilized pre-established grid labels in field experiments (each experimental plot, row, and column has a unique identification number). During data collection, the specific insertion position at the X-th node from the top was recorded for each leaf used in manual measurements.

### 2.2 Image data preprocessing

**Figure 2:**
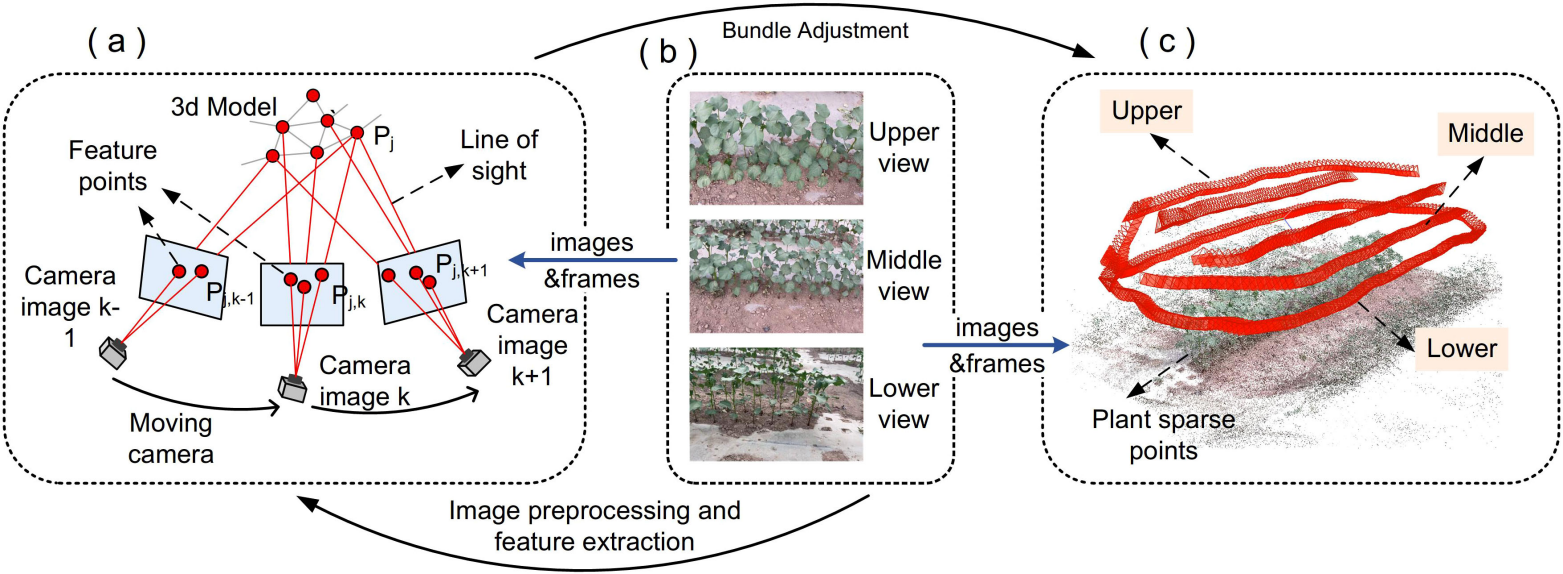
Process of sparse point cloud construction: **(a)** Spatial point cloud prediction based on adjacent images through camera pose estimation, triangulation, and bundle adjustment; **(b)** Schematic diagram of image acquisition, divided into upper, middle, and lower acquisition angles; **(c)** Sparse point cloud obtained via motion recovery techniques.

Preprocessing of the collected raw video data primarily involves analyzing the pixel proportion of target cotton and the frame overlap rate between adjacent frames. Images with insufficient target area coverage and data segments with excessively high frame overlap rates are discarded. Camera pose estimation and sparse point cloud reconstruction were achieved using pose and position recovery technology. The principle is shown in **Figure 2**.

### 2.3 Rapid 3D reconstruction technology for group crops

Three-dimensional reconstruction serves as a fundamental step in high-throughput phenotyping analysis of cotton fields, with modeling results directly impacting the reliability of trait quantification. Based on multi-view images captured, crop 3D reconstruction methods primarily include motion recovery techniques for multi-view stereo (MVS) (Pepe et al., 2022) and neural radiant fields (NeRF) (Mildenhall et al., 2021). Meanwhile, 3D Gaussian Splatter(3DGS) (Bao et al., 2025), an emerging scene rendering technique, has seen limited research in crop population modeling. This study evaluates the effectiveness of MVS, NeRF, and PhenotypeAI in crop population modeling using collected cotton canopy envelope data.

**Figure 3:**
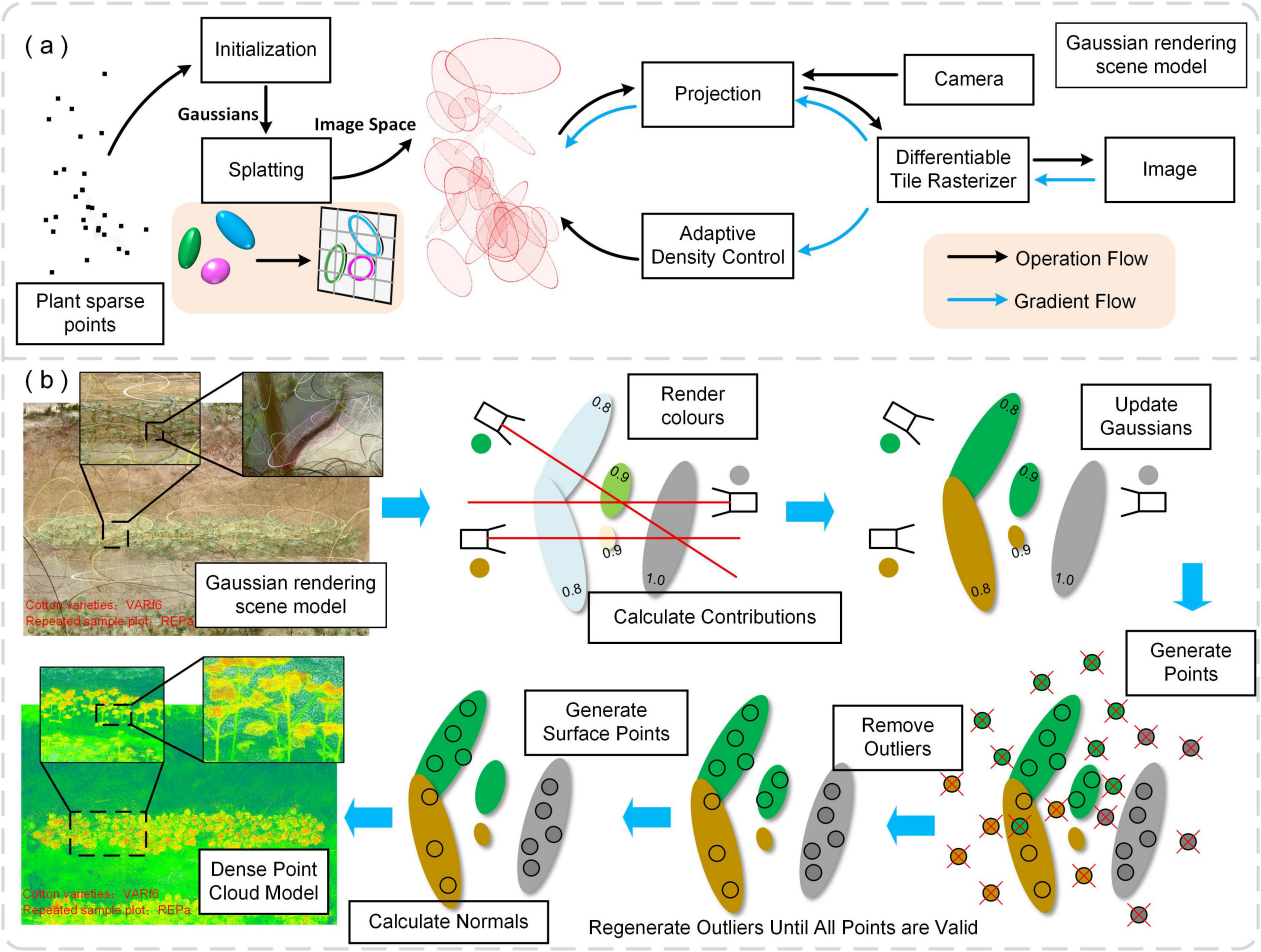
Three-dimensional Gaussian splatter and dense point cloud construction: **(a)** Initial Gaussian splatter is constructed, with adjustments to position, color, and transparency to achieve cotton cluster scene rendering; **(b)** Dense point cloud generation primarily involves Gaussian initialization and filtering, color rendering, point sampling, and outlier handling processes.

The technical implementation of 3D Gaussian modeling in cotton population reconstruction is essentially based on a synergistic mechanism of the four elements: geometric representation, differentiable rendering engine, adaptive optimization and dynamic topology regulation. The principle is shown in **Figure 3**. The mathematical basis stems from parameterizing the plant morphology as a collection of anisotropic Gaussian spheres (Equation 1). Each Gaussian cell defines its spatial distribution characteristics through the center position *µ*, the covariance matrix Σ, where Σ is parameterized by the rotation matrix R and the scaling matrix S to capture the organ-level growth characteristics of cotton, such as leaf blade stretching along the leaf vein direction, and stem geometry characteristics.

(1) $$G(x)=\text{exp }(-\frac{1}{2}{\left(x-\mu \right)}^{T}{\text{Σ}}^{-1}(x-\mu ))$$

where *μ* and Σ are the Gaussian center position and the covariance matrix, respectively, the latter of which mainly serves to control the shape and direction of the Gaussian distribution. The covariance matrix is decomposed by the rotation matrix R and the scaling matrix S as Equation 2:

(2) $$\text{Σ}=\text{RS}{\text{S}}^{T}{\text{R}}^{T}$$

where S denotes each axial scaling factor and R is the rotation matrix derived from the quaternion *q*. Based on this, the differentiable Gaussian sputtering technique projects the 3D Gaussian to the 2D image plane and establishes a bridge from parameter space to pixel space. The process achieves 3D to 2D geometric mapping through the camera projection matrix *K* with the view transformation *W* and calculates the color contribution of each pixel using the radial basis function property of the Gaussian kernel. And the projected covariance matrix Σ' is computed from the camera internal reference matrix *K* and the view transformation *W* with the core equation of Equation 3:

(3) $${\text{Σ}}^{\prime }=(\text{JW}\Sigma {\text{W}}^{T}){\text{J}}^{T}$$

where *J* is the Jacobi matrix of the perspective projection. The output of the above differentiable pipeline drives the convergence process of the optimization objective function. The objective function consists of an L1 luminance loss, which is a direct measure of pixel-level color differences, weighted by a structural similarity loss (SSIM), and a structural similarity loss (SSIM), which enhances the sensitivity of the optimization to the details of the edges of the plant by comparing the luminance, contrast, and structural information within an image block.

### 2.4 Instance segmentation method for plant point cloud ensembles

This study proposes an instance segmentation strategy for cotton field point clouds, which primarily encompasses three integral components: preprocessing of cotton field scene point clouds, extraction of core points within plant localization zones, and spatially constrained segmentation of individual plant point clouds.

#### 2.4.1 Step one: preprocessing of cotton field scene point clouds

**Figure 4:**
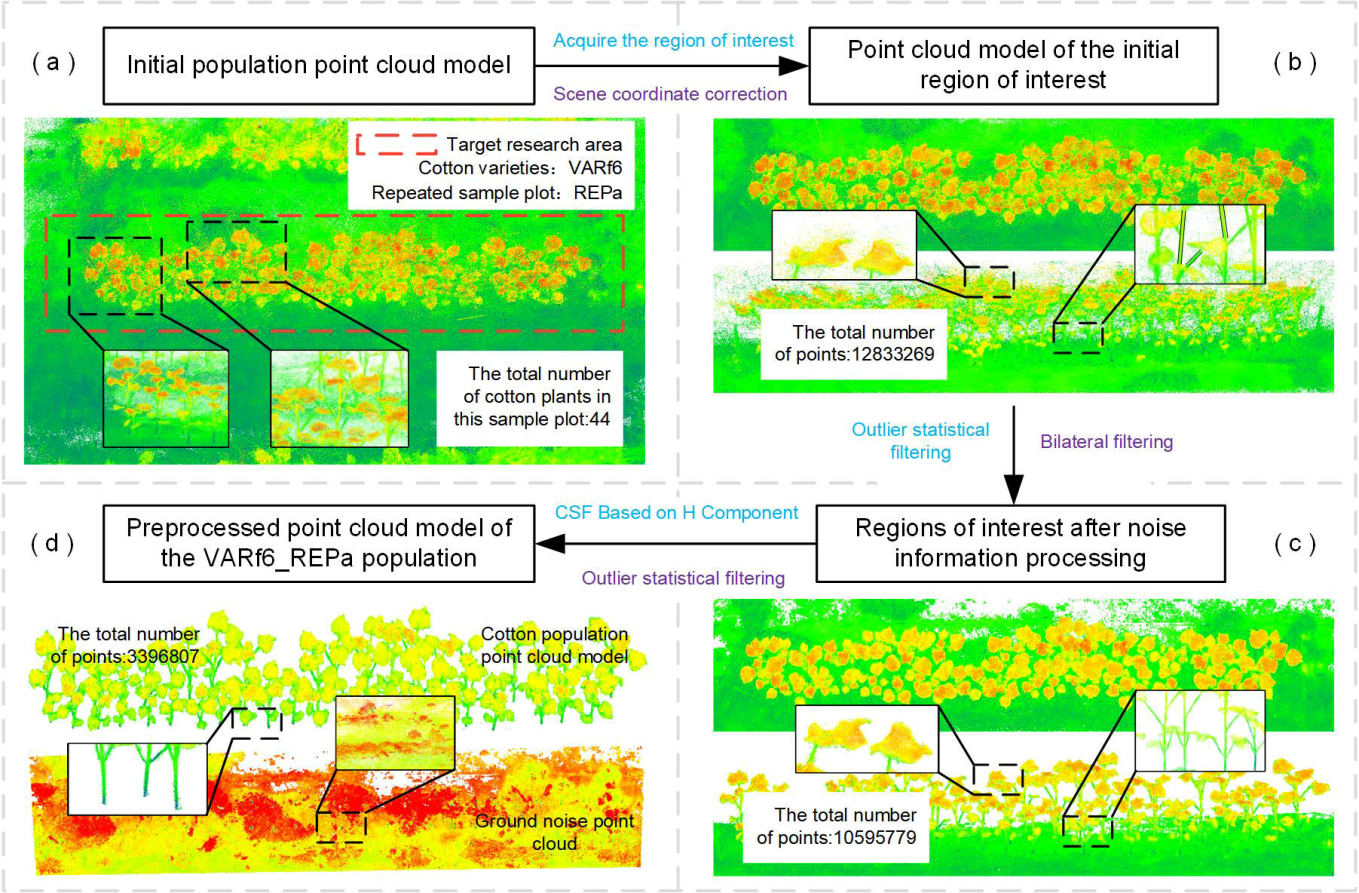
Preprocessing of cotton field scene point clouds. **(a)** Dense point cloud model of the cotton field constructed from the collective 3D reconstruction of VARf6 variety sample plot ‘a’. **(b)** Acquisition of the point cloud within the region of interest. **(c)** The region of interest point cloud after the application of statistical and bilateral filtering. **(d)** Implementation of an enhanced filtering methodology to facilitate the partial segregation of ground points **(GS)** from plant points (GP), followed by subsequent refinement processing of the GP component.

To extract crucial regions of interest from a large volume of extraneous background point cloud data, as depicted by the red box in **Figures 4a**, a coordinate transformation is initially applied to the point cloud within the region of interest. Perform coordinate transformation on the point cloud of the region of interest, adjusting the ground-level point cloud to a horizontal orientation to achieve coordinate correction for cotton’s upward growth.

Based on the RANSAC algorithm, ground points are detected and a plane is fitted, yielding a plane equation *ax* + *by* + *cz* + *d* = 0. In this equation, (*a, b, c*) represents the normal vector of the plane, and (*x, y, z*) denotes the coordinates of any point on the plane. The normal vector of the 242 ground plane is computed and its direction is corrected. This ground normal vector *n* is then rotated to align with *a* target vector *t*, and the corresponding rotation quaternion *q* is constructed as Equation 4:

(4) $$\text{q}=(w,x,y,z)=(\text{cos }\frac{\theta }{2},\text{sin }\frac{\theta }{2}\cdot k_{x},\text{sin }\frac{\theta }{2}\cdot k_{y},\text{sin }\frac{\theta }{2}\cdot k_{z})$$

where, $\text{k}=\frac{\text{n}\times \text{t}}{∥\text{n}\times \text{t}∥}$ is the axis of rotation, and θ=arccos (n·t) is the angle of rotation. The rotation matrix for global coordinate correction R is then calculated using the quaternion:

(5) $$\text{R}=[\begin{array}{ccc} 1-2y^{2}-2z^{2} & 2xy-2zw & 2xz+2yw\\ 2xy+2zw & 1-2x^{2}-2z^{2} & 2yz-2xw\\ 2xz-2yw & 2yz+2xw & 1-2x^{2}-2y^{2} \end{array}]$$

Applying this rotation matrix to the entire point cloud within the region of interest completes the coordinate transformation for the cotton canopy. Next, the Statistical Outlier Removal algorithm and bilateral filtering method were employed to eliminate outlier points within the neighborhood of plant point clouds and other non-plant points (i.e., noise signals) from the region of interest (**Figures 4b, c**). Finally, a ground filter was developed based on the Cloth Simulation Filter (CSF) method, which segmented the cotton point cloud within the region of interest into ground plants (GP) and ground surface (GS). While originally designed for monitoring and surveying extremely large ground structures, we discovered that converting the cotton point cloud color to the HSI color space revealed a significant distinction between cotton and the ground in the H component. Therefore, the filter was optimized by augmenting the point cloud’s H component and increasing its nodes, resulting in the HCSF (HSI-enhanced Cloth Simulation Filter). The filtered cotton plant point cloud is shown in **Figure 4d**.

#### 2.4.2 Step 2: extraction of core points for plant localization

**Figure 5:**
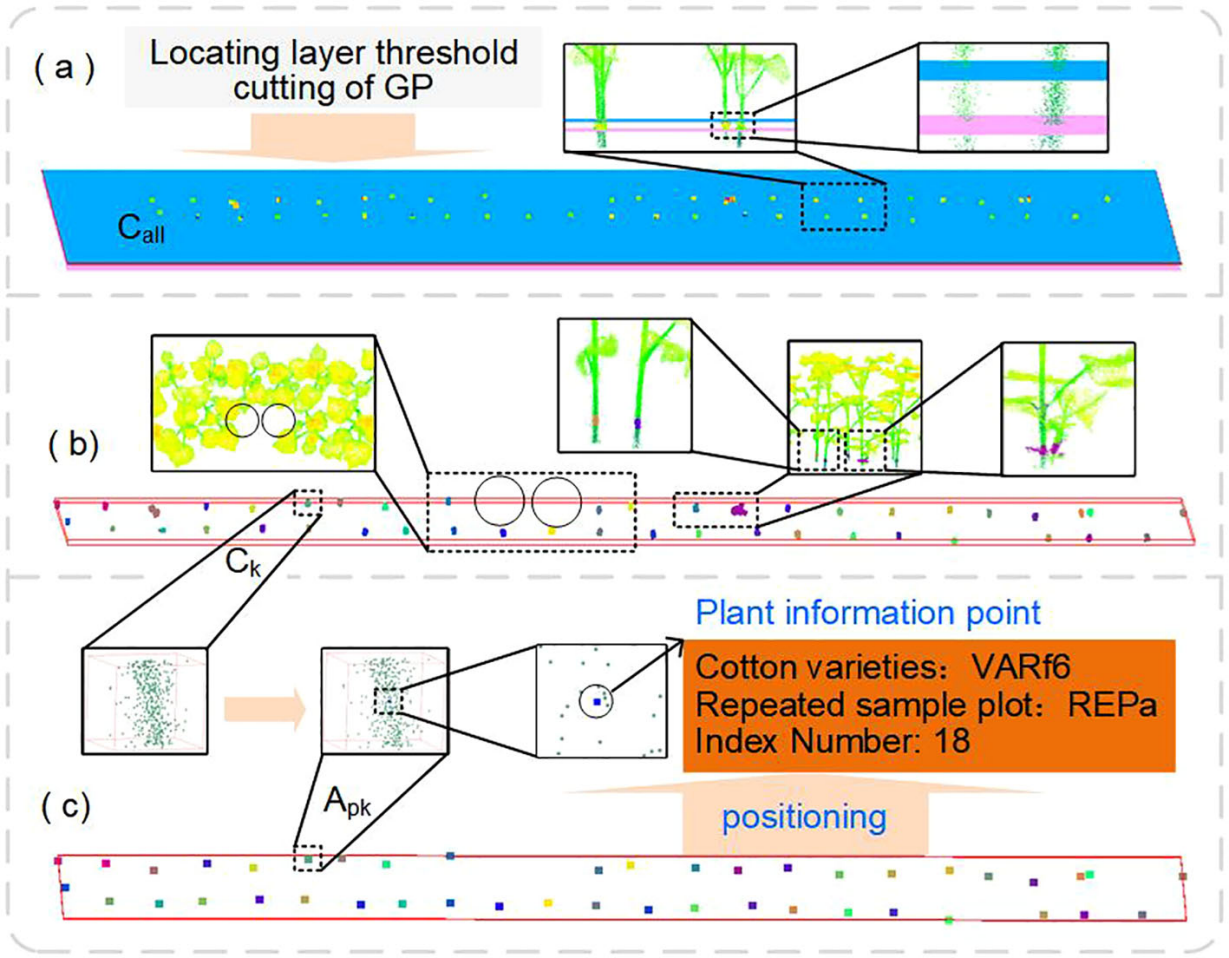
Extraction of core points for plant localization. **(a)** Point cloud subsetting by height layers. **(b)** Clustering of point clouds within the localization area to obtain plant localization point clouds, with different plants distinguished by random colors. **(c)** Obtaining the mean point at a fixed height within the localization area as the plant localization point, enabling plant information association.

Cotton canopy leaves tend to intersect as they compete for light resources, but the main stems naturally remain distinct. Therefore, specific regions of the cotton main stems can serve as localization areas for individual plants, enabling the calculation of a localization point for each plant (**Figure 5**). Unlike K-means, DBSCAN generates non-convex clusters based on the dataset and does not require the number of clusters as an input parameter. It only necessitates defining the minimum number of points (MinPts) or the neighborhood radius (*ϵ*-neighborhood of a point) required to form a cluster, thereby achieving clustering of arbitrarily shaped point clouds.

First, a localization height layer [*L_l_, L_h_*] is defined. From the cotton point cloud *P*, which has had ground and noise points removed, a subset of point clouds *C*_all_ is extracted. This subset represents the root localization point cloud collection for all plants. Next, independent plant root point clusters are formed. Starting from an arbitrary point *p* in the point cloud set *C*_all_, its *ϵ*-neighborhood (denoted as *Nε*(*p*)), as Equation 6 is defined as the set of all points *q* (including *p*) such that the distance between *p* and *q* is within the *ϵ* radius:

(6) $$N_{\in }(p)=\{q\in D|\text{dist}(p,q)\leq \epsilon \}$$

If the current neighborhood satisfies *|N_∈_*(*q*)*|≥* MinPts, a cluster is initiated. Otherwise, point *p* is classified as a noise point. This process is repeated for every point in *D*. When the point cloud exhibits a relatively uniform distribution, it is not necessary to provide both MinPts and *ϵ* parameters simultaneously. Therefore, *ϵ* can be determined using the following formula:

(7) $$\epsilon =\sqrt{\frac{T\cdot \text{MinPts}\cdot \text{Γ}[(\frac{1}{2})\cdot n+1]}{m\sqrt{\pi^{n}}}}$$

Where *m* represents the number of points, *n* is the dimensionality of the points, Γ is the Gamma function, *X* is the point cloud set, and *T* is the volume of the space formed by *m* points (Equation 8). The clustering result yields *s* point clusters: *C*_1_*, C*_2_*,…,C_s_*. Each cluster corresponds to the root localization region of a single cotton plant. Finally, the mean point of cluster *C_k_*at a fixed height *L_h_*is calculated, serving as the localization point *A_pk_* (Equation 9) for cluster *C_k_*:

(8) $$T=\prod_{i=1}^{n}\{\text{max }(X_{i})-\text{min }(X_{i})\}$$

(9) $$A_{pk}=(\frac{1}{\left|C_{k}\right|}\sum_{p\in C_{k}}x_{p},\frac{1}{\left|C_{k}\right|}\sum_{p\in C_{k}}y_{p},{\bar{z}}_{0})$$

Where, ${\bar{z}}_{0}=L_{h}$. This process uniquely defines each cotton plant in the population by a localization point *A_pk_*, which serves as its identification code. This identification code, together with the plant variety, plot number, plant index number, and spatial coordinates, constitutes the field information for each plant. Subsequently, a mapping relationship is established with the extracted phenotypic information.

#### 2.4.3 Step 3: spatially constrained plant point cloud segmentation

Region growing is a classic algorithm in point cloud segmentation. Its main idea is to evaluate the similarity between seed points and neighboring points, classifying units with similar characteristics into one category. Its core steps involve seed point selection, similarity metric construction, and cluster scope definition. Envelope analysis of cotton plants reveals that the angle between their main stem and the outermost points of the leaf canopy consistently falls within a certain range. This study uses the point cloud set of the plant’s localization region as initial seed points. By calculating spatial constraint features based on plant height and plant architecture, it enables the growth of the population point cloud within the constrained space of each plant. This approach differs from previous region growing methods by innovatively linking the scope of region growing with the plant’s own characteristics such as height and architecture, thereby improving the algorithm’s efficiency and accuracy.

It primarily includes four steps: calculation of plant region height *R_hk_*, definition of spatial constraint range *C_yk_*, construction of multi-constraint growth rules, and correction of overlapping point cloud attribution. The pseudo-code of the point cloud instance segmentation step is presented in **Algorithm 1**.

Algorithm 1

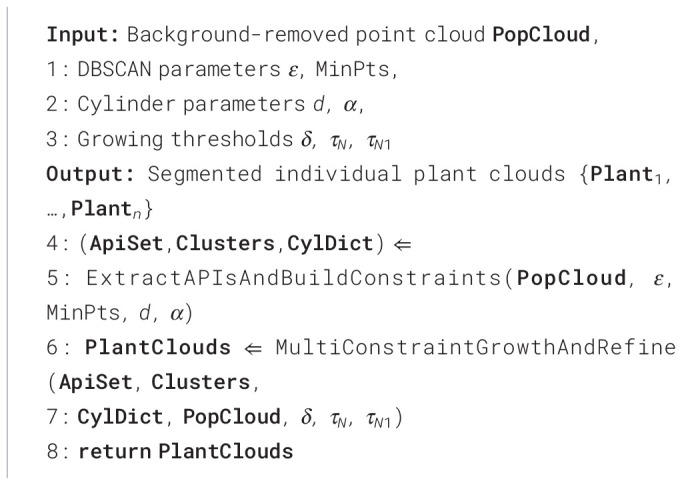

1. Calculation of plant region height *R_hk_*.

Centered at each plant localization point *A_pk_*, a cylindrical envelope with radius *d* is constructed. The point cloud *P_k,_*_small_ within this cylinder is then obtained, such that:

(10) $$R_{hk}=\text{max }\{z_{p}|p\in P_{k,\text{small}}\}$$

2. Definition of spatial constraint range *C_yk_*.

Defining a spatial constraint range *C_yk_*for each plant limits the growth scope of its point cloud, significantly reducing the number of point clouds within that growth range. This prevents overgrowth and minimizes erroneous segmentation of point clouds between adjacent plants.

Centered at the plant localization point *A_pk_*, and constrained by the angle between the plant’s main stem and the outermost envelope point, the spatial constraint range *α* for the plant point cloud is constructed as follows:

(11) $$C_{yk}:\{\begin{array}{l} {\left(x-A_{pk}\times x\right)}^{2}+{\left(y-A_{pk}\times y\right)}^{2}\leq r_{k}^{2}\\ z\geq z_{\text{min}} \end{array}$$

Where, *r_k_*= *R_hk_ ·* tan(*α*) is the base radius of the spatial constraint, and *z*_min_ represents the minimum Z-coordinate of the point cloud within the region of interest. The pseudo-code for the calculation of plant positioning points and growth radius is shown in **Algorithm 2**.

Algorithm 2

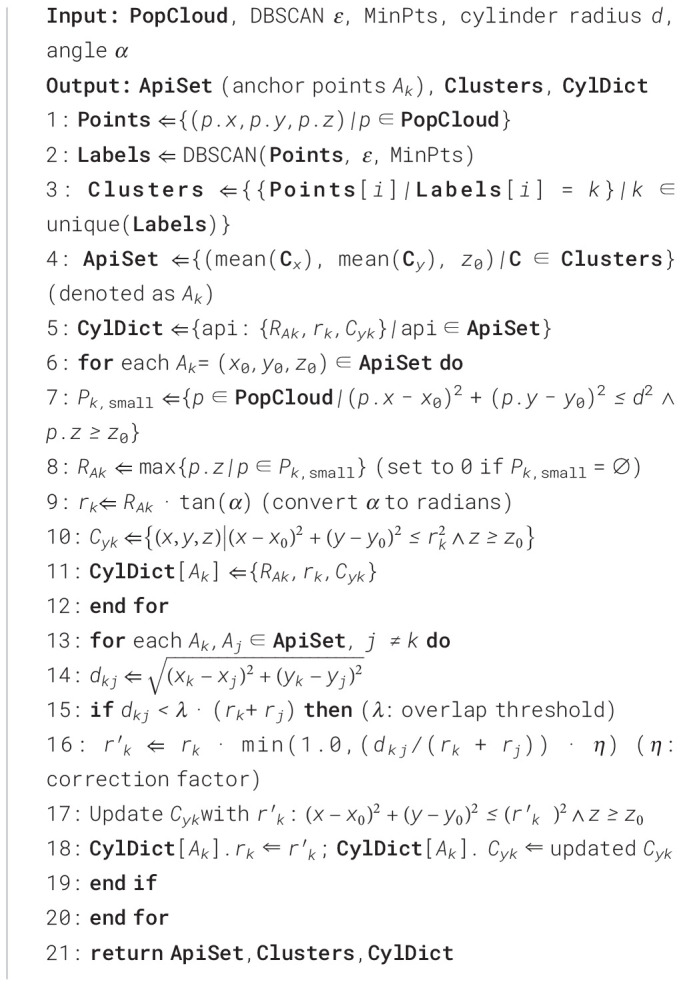

3. Construction of multi-constraint growth rules.

We define three constraint metrics: the Euclidean distance constraint $D_{\left(p,q\right)}$ the normal vector similarity constraint $N_{\left(p,q\right)}$, and the spatial range constraint $Y_{\left(q,C_{yk}\right)}$. Their respective formulas are as follows:

(12) $$D_{\left(p,q\right)}=\{\begin{array}{ll} 1 & ||p-q||<\delta \\ 0 & \text{otherwise} \end{array}$$

(13) $$N_{\left(p,q\right)}=\frac{{\text{n}}_{p}\cdot {\text{n}}_{q}}{\left||{\text{n}}_{p}||\cdot ||{\text{n}}_{q}|\right|}\geq \tau_{N}$$

(14) $$Y_{\left(q,C_{yk}\right)}=\{\begin{array}{ll} 1 & q\in C_{yk}\\ 0 & \text{otherwise} \end{array}$$

Where, δ represents the distance threshold between growing points, ensuring the continuity of the point cloud space; $\tau_{N}$ is the cosine of the angle between normal vectors; and ${\text{n}}_{p}$ and ${\text{n}}_{q}$ are the unit normal vectors of points p and q, respectively. For a point p in the queue, the growth condition for its neighboring point q is $\left(D_{\left(p,q\right)}=1)\cap (N_{\left(p,q\right)}\geq \tau_{N})\cap (Y_{\left(q,C_{yk}\right)}=1\right)$.

4. Correction of overlapping point cloud attribution.

The overlapping regions of the constraint ranges invariably consist of point clouds from the marginal leaves of adjacent plants. The ownership of these intersection points is determined by the leaves within their respective neighborhood spaces. In a spatial context, this manifests as continuous local point cloud clusters, characterized by high density and consistent normal vectors within their neighborhoods. Extract the local neighborhood point set $N_{kq}=\{p\in {\text{P}}_{k}|||\text{p}-\text{q}||<R_{\text{loc}}\}$for the intersection point q within the point cloud *P_k_*of candidate plant *k*. Subsequently, filter out points belonging to different leaves based on normal vector consistency to obtain the fitting point set *F_k,q_*. The RANSAC algorithm is then applied to *F_k,q_*to fit a plane, whose equation is:

(15) $$a_{k,q}x+b_{k,q}y+c_{k,q}z+d_{k,q}=0$$

Where $\left(a_{k,q},b_{k,q},c_{k,q}\right)$ is the unit normal vector of the plane, satisfying $a_{k,q}^{2}+b_{k,q}^{2}+c_{k,q}^{2}=1$. The intersection points are then verified for assignment based on local plane distance and curvature consistency. The distance $d_{k,q}$ from the intersection point q to this plane can be defined as:

(16) $$d_{k,q}=\frac{\left|a_{k,q}x_{q}+b_{k,q}y_{q}+c_{k,q}z_{q}+d_{k,q}\right|}{\sqrt{a_{k,q}^{2}+b_{k,q}^{2}+c_{k,q}^{2}}}$$

Calculate the mean curvature $\mu_{k,q}$ and standard deviation $\sigma_{k,q}$ of $F_{k,q}$. The curvature $\kappa_{q}$ of q should satisfy $|\kappa_{q}-\mu_{k,q}|\leq \gamma_{1}\sigma_{k,q}$, while also satisfying the local planar distance $d_{k,q}<\gamma_{2}$. Where, $\gamma_{1}$ and $\gamma_{2}$ are empirical values. If these conditions are met, the intersection point q is assigned to plant k. The pseudo-code of the region growing step based on dynamic constraints is shown in **Algorithm 3**.

Algorithm 3

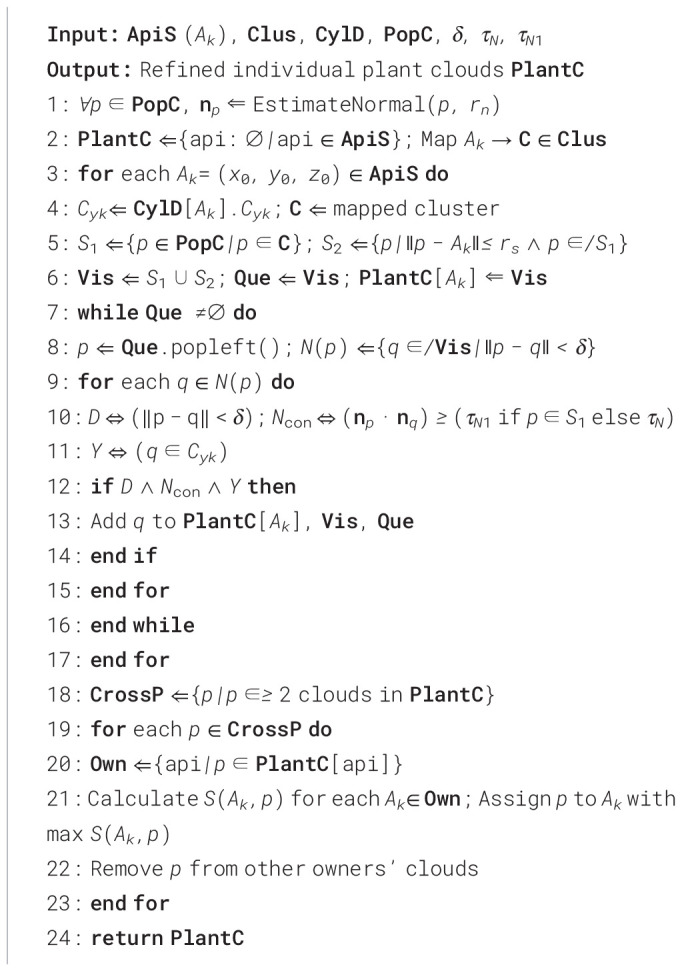

### 2.5 Phenotypic trait analysis

Cotyledonary Node Height (FCNH) in cotton is a crucial morphological indicator for assessing lodging resistance and early growth vigor in cotton seedlings. Excessively high FCNH increases the risk of plant lodging. Furthermore, studies have demonstrated a direct correlation between the number of main stem nodes (including cotyledonary nodes) and boll distribution and yield (Reddy et al., 2017). Its mathematical expression is:

(17) $$\text{FCNH}=z_{\text{fn}}-z_{\text{gd}}$$

Where *z*_fn_ represents the Z-coordinate of the cotyledonary node, and *z*_gd_ represents the ground datum plane. The cotyledonary node is identified by first removing leaf sections from the cotton plant using plane fitting, then obtaining the intersection points of the main stem and branches through line fitting. The intersection point with the minimum Z-coordinate is designated as the cotyledonary node.

Plant height (PH) is defined as the vertical distance from the cotyledon node to the growing point at the apex of the main stem. It serves as a core morphological parameter reflecting the overall growth posture of the plant. PH is a crucial indicator for monitoring cotton growth and assessing lodging resistance (Reddy et al., 2017). Additionally, in breeding selection, consistency in plant height is a key criterion for evaluating hybrid cotton varieties. Its mathematical expression is:

(18) $$\text{PH}=z_{\text{msv}}-z_{\text{gd}}$$

Where, *z*_msv_ represents the Z-coordinate value of the plant’s highest point. Leaf area refers to the sum of the surface areas of individual leaves on a plant, significantly influencing crop photosynthetic efficiency and stress response. For pre-processed plant point clouds, leaf point clouds are first obtained through plane fitting. Subsequently, cluster segmentation is performed based on normal and curvature features. Poisson surface reconstruction is then applied to the clustered point clouds, and the surface area of the resulting Poisson surface represents the leaf area of that particular leaf.

### 2.6 Evaluation measure

The experiment evaluates the 3D modeling process in terms of image fidelity, perceptual quality and point cloud sampling. Peak Signal-to-Noise Ratio (PSNR) is based on the mean square error and is mainly implemented to quantify the signal fidelity of the reconstructed image with the mathematical formula:

(19) $$\text{PSNR}(I,K)=10\log_{10}(\frac{{\text{MAX}}_{\text{I}}^{2}}{\text{MSE}})$$

(20) $$\text{MSE}=\frac{1}{m\times n}\sum_{i=1}^{m}\sum_{j=1}^{n}{\left(I(i,j)-K(i,j)\right)}^{2}$$

Where, *I*(*i,j*) and *K*(*i,j*) respectively denote the pixel values of the original and reconstructed images. The Structural Similarity Index Measure (SSIM) takes brightness, contrast and structural similarity into account to assess whether the reconstructed scene has structural features similar to the real scene with the mathematical formula:

(21) $$\text{SSIM}(x,y)={\left[l(x,y)\right]}^{\alpha }\cdot {\left[c(x,y)\right]}^{\beta }\cdot {\left[s(x,y)\right]}^{\gamma }$$

Where, *x* and *y* denote localized window blocks extracted from two distinct images. Specifically, *l*(*x,y*) quantifies the luminance similarity, *c*(*x,y*) characterizes the contrast similarity, and *s*(*x,y*) measures the structural similarity, with *α, β, γ* functioning as a weighting parameter. LPIPS (Learned Perceptual Image Patch Similarity) measures the difference between rendered images and real images in the depth feature space, better reflecting the perceptual quality of generated images than traditional metrics:

(22) $$\text{LPIPS}(I,K)=\sum_{l=1}w_{l}\cdot ∥F_{l}(I)-F_{l}(K){∥}_{2}^{2}$$

Where $w_{l}$ represents the weighting layer, $F_{l}$ denotes the *l*-th layer features extracted from the pre-trained network, and $||\cdot {\left|\right|}_{2}^{2}$ signifies the squared Euclidean distance between the corresponding features. To evaluate the uniformity of distribution in the reconstructed cotton population point cloud, the point cloud surface density (Equation 23) and volume density (Equation 24) metrics were selected:

(23) $$D_{\text{sur}}=\frac{N}{A_{cs}}$$

(24) $$D_{\text{vol}}=\frac{N}{V_{cv}}$$

where *A*_cs_ is the surface area of the convex packet of the point cloud (mm^2^), *V*_cv_ is the volume of the convex packet (mm^3^), and *N* is the effective number of points.

In Cloudcompare software, plant point clouds were manually annotated from the group point cloud, with each plant assigned a distinct label. This serves as ground truth (GT) data for evaluating the accuracy of instance segmentation algorithms. In the evaluation of plant point cloud segmentation, if a cotton plant exists and is segmented from the point cloud, it is called true positive (TP); if a cotton plant does not exist but is segmented from the point cloud, or a plant exists, it is completely segmented and a part of the point cloud of the nearby cotton is also segmented into the plant, it is called false positive (FP); if a cotton is not segmented and assigned to a nearby cotton, or a cotton exists and is segmented, but the segmentation is incomplete, the part of the cotton point cloud is identified as the part of other plants, which is called false negative (FN). High TP values and low FN and FP values correspond to high accuracy. Recall rate (r), intersection-union ratio (IoU), precision rate (p) and F score are calculated, which are commonly used to evaluate the segmentation performance of point cloud instances (Tao et al., 2015).

### 2.7 Parameter sensitivity analysis and robustness verification

To ensure the generalization and robustness of the proposed instance segmentation method, this study follows a systematic parameter tuning and validation pipeline. All core algorithm parameters were optimized via grid search combined with manual visual assessment on a representative standardized experimental plot (VARf6REPa). This tuning set was used to identify parameter combinations that achieve an optimal balance between clustering completeness, boundary clarity, and other visual quality metrics and quantitative indicators.

The core parameters determined through tuning are listed in **Table 2**. These parameter values are set based on typical geometric scales of cotton plants in standardized breeding fields: the neighborhood parameter *∈* is smaller than the typical inter-plant spacing to distinguish adjacent plants, the distance threshold *δ* matches the branch diameter scale, and the normal vector threshold *τ_N_*is used to capture continuous orientation of plant surfaces.

**Table 2:** Core parameters for instance segmentation.

Parameter	Range	Optimized value
DBSCAN neighborhood *ϵ*	0.8 cm – 2.0 cm	1.2 cm
Minimum points *MinPts*	3 – 10	6
Initial cylinder radius *d*	2.0 cm – 4.0 cm	3.0 cm
Search cone angle *α*	20*^°^* – 50*^°^*	35*^°^*
Distance threshold *δ*	1.0 cm – 3.0 cm	1.5 cm
Normal vector threshold *τ_N_*	0.70 – 0.95	0.85

The optimized parameter set was fixed and directly applied to eight additional independent trial plots covering different varieties and planting replicates for validation. The method achieved consistently high segmentation accuracy across all test plots (average F1-score of 91.32%). The results demonstrate that the parameter group tuned based on representative samples exhibits strong generalization capability in the standardized breeding field environment, and the proposed method is robust to common field variations such as plant density fluctuations and point cloud quality changes.

To evaluate the robustness of the method, we tested the impact of key parameters varying within ±20% of their nominal values on segmentation accuracy (mIoU). The results show that variations in all parameters within this range led to less than a 5% change in mIoU, indicating the method’s insensitivity to parameter settings. Furthermore, tests were conducted on subsets of data with different reconstruction qualities. Segmentation accuracy (both mIoU and F1-score) remained stable across all subsets, verifying the robustness of the method to common field variations.

## 3 Results

The experimental setup was equipped with an Intel Core i9-14900F processor, 128 GB of RAM, and an NVIDIA GeForce RTX 4090 24G graphics card. The system operated on Ubuntu 20.04, where COLMAP was employed for feature extraction and sparse reconstruction. The environment for 3D Gaussian Splatting and dense point cloud reconstruction was configured with CUDA 11.8 and PyTorch 2.1.1. Subsequent point cloud processing was also conducted on Ubuntu 20.04, primarily utilizing the PCL library, along with Python Open3D and NumPy for implementation.

### 3.1 3D reconstruction of cotton plant populations

**Figure 6:**
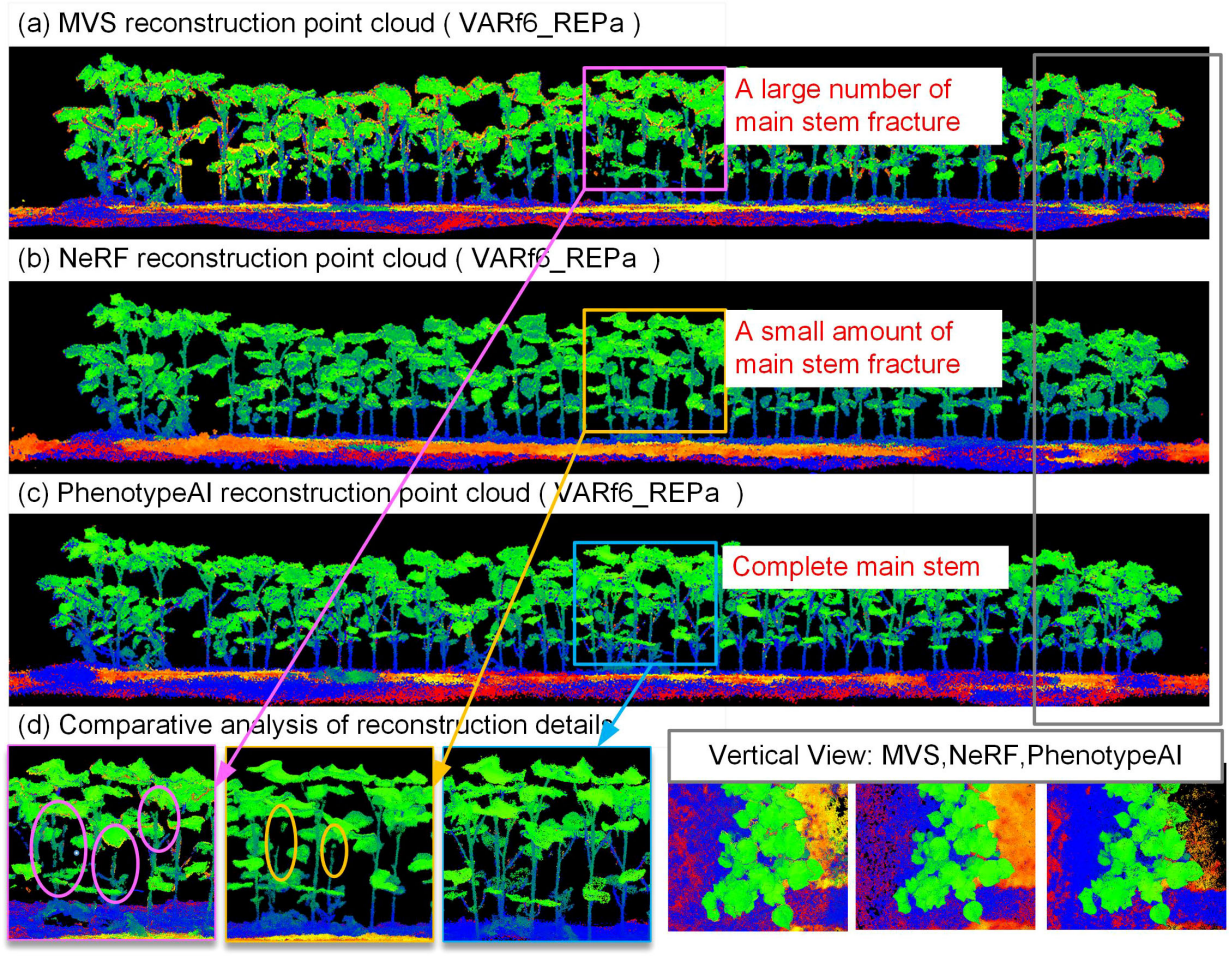
Comparison of VARf6_REPa population modeling methods. **(a)** Group point cloud constructed using the MVS method; **(b)** Group point cloud constructed using the NeRF method; **(c)** Group point cloud constructed using the PhenotypeAI method; **(d)** Local enlargements and partial top-down views of group point clouds generated by the three methods.

Three-dimensional modeling of cotton populations constitutes the foundational and paramount initial phase for the extraction of cotton population phenotypic traits. The experiment selected MVS, NERF, and PhenotypeAI methods to model cotton populations based on the constructed cotton field dataset. The experimental outcomes underwent qualitative scrutiny, focusing on the completeness of reconstruction for various cotton cultivar sample plots and the incidence of voids or gaps within plant organs. **Figure 6** illustrates the three-dimensional point cloud models derived from the MVS, NeRF, and PhenotypeAI, taking the VARf6_REPa group data as an example. **Table 3** provides a quantitative analysis, detailing the computational time expended for modeling and elucidating key performance metrics related to accuracy. At the population scale, all three methods—MVS, NeRF, and PhenotypeAI—achieved plant-level modeling of cotton. The red areas along the plant edges in the top view reveal significant artifact points in the MVS model. Delving into the scale of individual plant organs, panel (d) distinctly illustrates that the main stems reconstructed by both MVS and NeRF methods exhibit considerable voids, and in some instances, complete point cloud omissions. Conversely, the top-down perspective provided in panel (d) indicates that all three approaches attained commendable reconstruction of cotton leaf point clouds within the plant canopy. Consequently, the PhenotypeAI method exhibits less noise and more continuous plant organs in the point cloud reconstruction of cotton populations in the field.

**Table 3:** Cotton population reconstruction accuracy analysis.

Varieties	Method	PSNR*↑*	LPIPS*↓*	SSIM*↑*	Time/h	Dsur	Dvol
VARc94	MVS	–	–	–	9.3	485	267
	NeRF	27.0	0.22	0.72	13.5	197	165
	PhenotypeAI	30.0	0.20	0.84	1.1	394	260
VARf6	MVS	–	–	–	8.5	549	291
	NeRF	27.8	0.24	0.80	14.2	220	183
	PhenotypeAI	31.3	0.18	0.86	1.2	437	309
VARx36	MVS	–	–	–	9.1	622	348
	NeRF	25.2	0.26	0.67	15.0	244	196
	PhenotypeAI	32.1	0.17	0.87	1.4	475	334

Regarding image quality evaluation metrics, the PSNR value for the VARc94 variety reached 30.0 dB, representing an 11.1% improvement over the NeRF method. The SSIM metric achieved 0.84, a 16.7% increase over NeRF, indicating that PhenotypeAI holds a significant advantage in preserving structural similarity. For the LPIPS perceptual quality metric, PhenotypeAI reduced the value for the VARx36 variety to 0.17, a 34.6% decrease compared to NeRF, demonstrating that its reconstruction results better align with human visual perception characteristics. Multi-variety comparisons show that the VARx36 variety achieved a PSNR of 32.1 dB in the PhenotypeAI model, 6.7% and 4.2% higher than VARc94 and VARf6 respectively.

In terms of computational efficiency, the PhenotypeAI model’s training time metrics further highlight its engineering value. Point cloud modeling requires only 1.1-1.4 hours, representing a reduction of over 90% compared to NeRF. This efficiency gain stems from PhenotypeAI’s adaptive Gaussian sphere optimization, which concentrates computational resources on regions of high feature significance. This achieves a surface point density of 475 points per square centimeter across the VARx36 variety, marking a 94.7% improvement over NeRF.

The study evaluated the performance differences of three modeling methods—MVS, NeRF, and PhenotypeAI in the 3D reconstruction of cotton plant populations. Experimental data indicate that the PhenotypeAI method demonstrated significant advantages in terms of visual quality, computational efficiency, and model accuracy.

**Figure 7:**
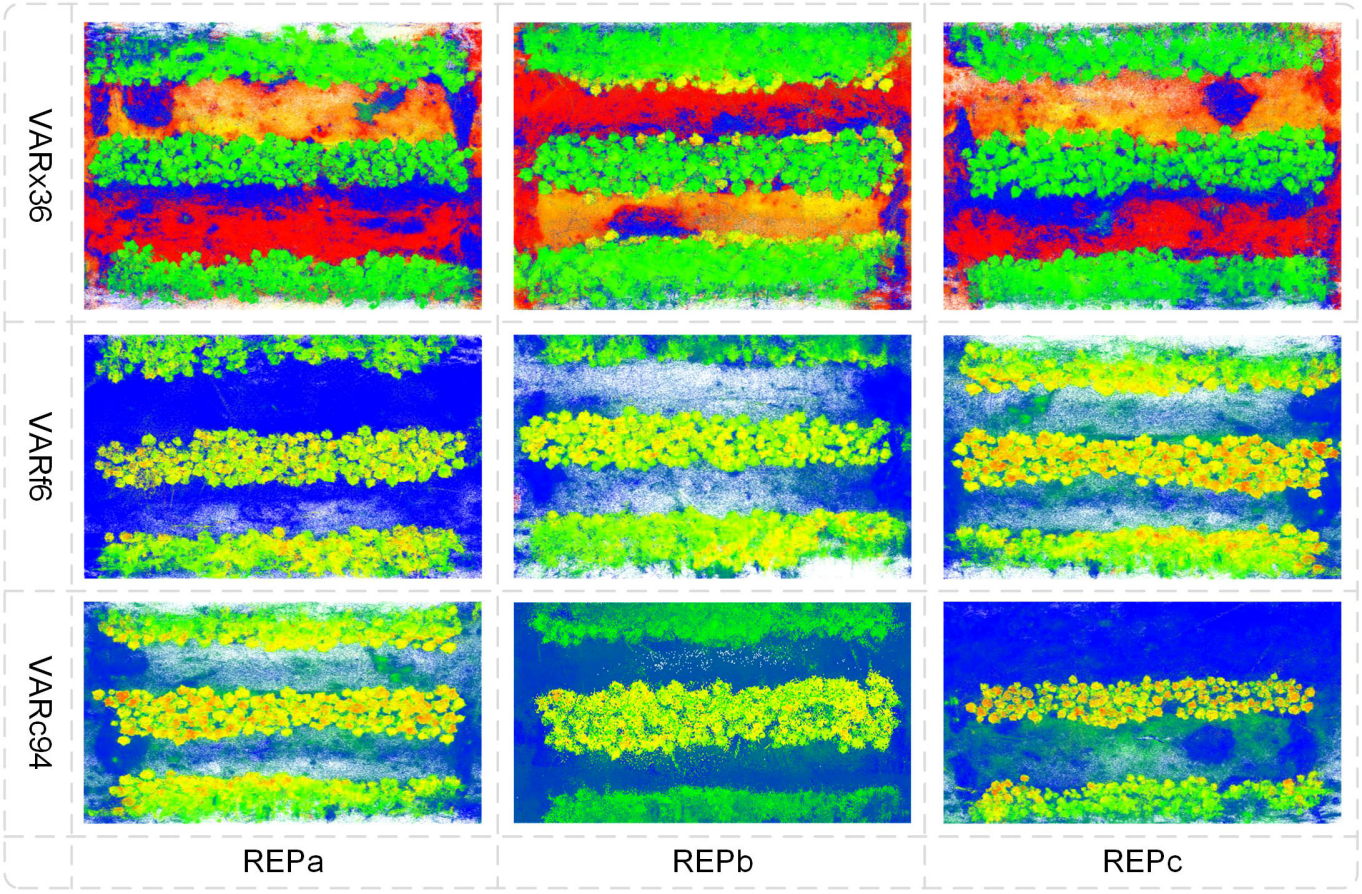
Cotton population reconstruction results. The varieties are VARx36, VARf6, and VARc94, with three experimental plots for each variety.

The plot point cloud of the sample area reconstructed using the PhenotypeAI method is shown in **Figure 7**. Based on this reconstructed point cloud, subsequent research on instance segmentation of cotton field plant point clouds will be conducted.

### 3.2 Point cloud processing and instance segmentation

**Figure 8:**
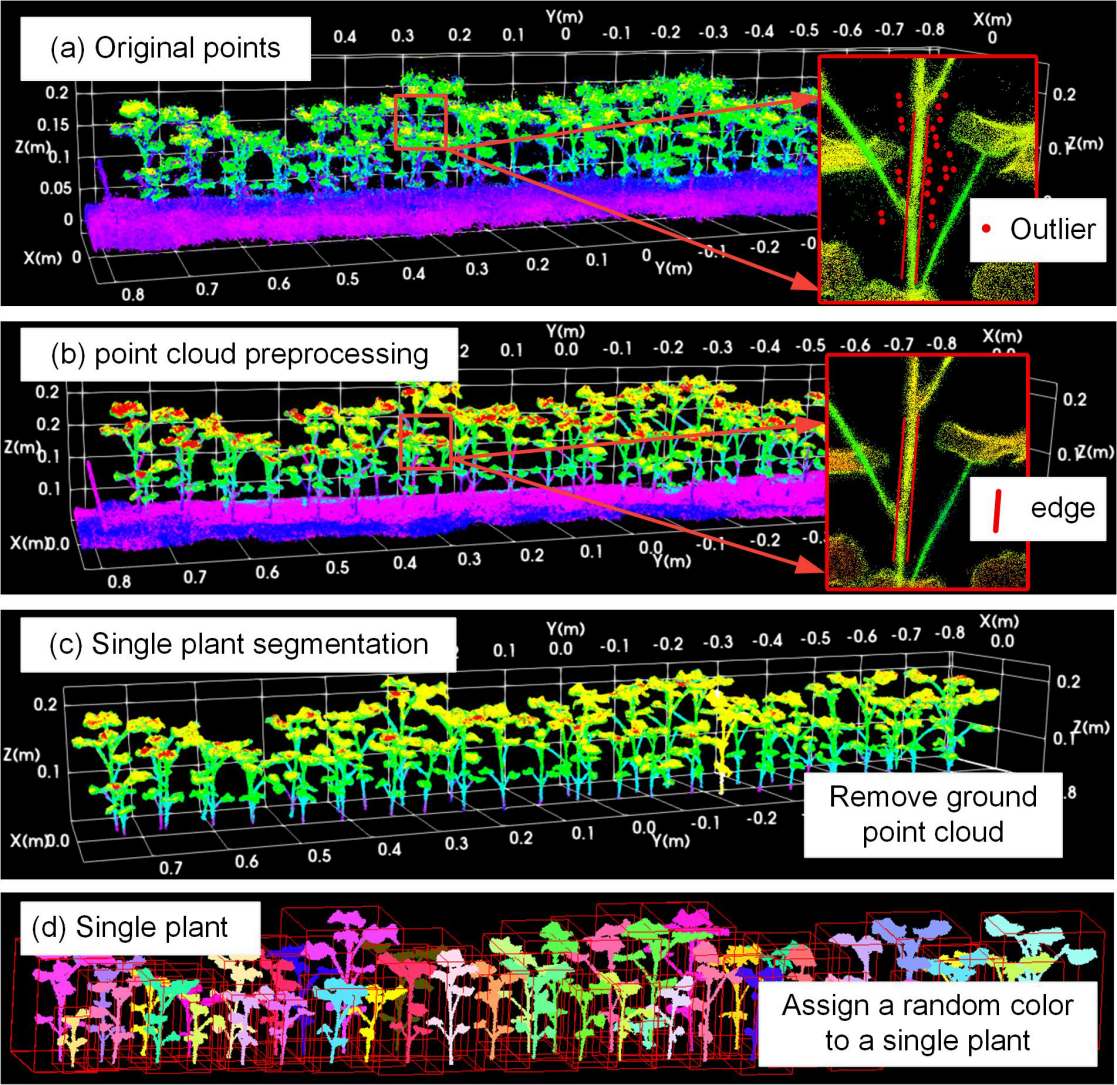
VARf6_REPa population point cloud instance segmentation results: **(a)** is the reconstructed original population point cloud, **(b)** is the population point cloud after preprocessing and noise removal, **(c)** is the cotton population point cloud after ground removal, and **(d)** is the segmented plant point cloud, with different individual plants represented by random colors.

The quantitative evaluation results of cotton population instance segmentation are shown in **Tables 4**, **5**. Combined with the overall preprocessing effect of point cloud, the integrity of segmented plants and the characteristics of noise distribution shown in **Figure 8**, the qualitative analysis is carried out. The qualitative analysis takes the VARf6_REPa group as an example.

**Table 4:** PhenotypeAI instance segmentation and optimization results.

Varieties	REP	Manually counted	Divided	FP	FN	TP	r	p	F
VARc94	a	43	43	3	5	40	88.89	93.02	90.91
	b	45	45	4	3	41	93.18	91.11	92.13
	c	45	45	5	5	40	88.89	88.89	88.89
VARf6	a	44	44	3	4	41	91.11	93.18	92.13
	b	43	43	3	4	40	90.91	93.02	91.95
	c	45	45	4	3	41	93.18	91.11	92.13
VARx36	a	48	48	5	3	43	93.48	89.58	91.49
	b	46	46	4	3	42	93.33	91.30	92.31
	c	44	44	4	5	40	88.89	90.91	89.89

**Table 4** records the evaluation results of the PhenotypeAI method on the segmentation accuracy of VARc94, VARf6 and VARx36 at the plant scale of each sample area of the three varieties. Among them, Manually calculated is the real number of cotton plants, Divided is the number of cotton plants detected by the algorithm, and the number of cotton plants in each sample area is correctly detected, mainly due to the fact that we determine the number and location of cotton plants through high-precision cotton positioning area.

**Table 5** records the evaluation results of the instance segmentation efficiency of the PhenotypeAI method on the plant scale of VARc94, VARf6 and VARx36, which is the total instance segmentation time of the population point cloud in each sample area and the average segmentation time of each plant.

**Table 5:** Time efficiency assessment of cotton population point cloud instance segmentation via PhenotypeAI.

Varieties	VARc94			VARf6			VARx36		
	a	b	c	a	b	c	a	b	c
*T*_tol_/s	56.22	57.18	60.94	58.12	56.40	60.15	62.09	61.38	58.42
*T*_avg_/s	1.31	1.27	1.35	1.32	1.31	1.34	1.29	1.33	1.33

As shown in **Table 6**, this study conducted a comparative evaluation of the proposed point cloud segmentation method against classical point cloud segmentation methods on the collected cotton plant point cloud dataset. The results demonstrate that our proposed point cloud segmentation method achieves superior performance across all segmentation accuracy metrics, significantly outperforming classical point cloud segmentation methods. Our method successfully identified all plant instances, owing to the proposed point cloud localization regions. This approach reduces over-reliance on crop morphological features and point cloud density during segmentation, achieving a maximum F-score of 92.08%.

**Table 6:** Comparison of results from different point cloud segmentation methods.

Method	Varieties	Manually counted	Divided	FP	FN	TP	r	p	F
DBSCAN	VARc94	133	79	41	36	38	51.35	48.10	49.67
	VARf6	132	81	37	29	44	60.27	54.32	57.14
	VARx36	138	75	43	35	32	47.76	42.67	45.07
Euclidean	VARc94	133	87	39	42	48	53.33	55.17	54.24
	VARf6	132	95	43	39	52	57.14	54.74	55.91
	VARx36	138	93	40	43	53	55.21	56.99	56.08
HDBSCAN	VARc94	133	112	21	20	91	81.98	81.25	81.61
	VARf6	132	115	18	17	98	85.22	84.48	84.85
	VARx36	138	118	22	19	99	83.90	81.82	82.85
Ours	VARc94	133	133	12	13	121	90.30	90.98	90.64
	VARf6	132	132	10	11	122	91.73	92.42	92.08
	VARx36	138	138	13	11	125	91.91	90.58	91.24

We recorded the segmentation efficiency results of the comparative algorithms, as shown in **Table 7**. The Euclidean method required the longest processing time for group point cloud segmentation, while the DBSCAN method was the fastest. However, DBSCAN failed to fully capture all cotton plants within the region. HDBSCAN, a more advanced geometric clustering baseline method, can automatically determine the number of clusters and handle density variations, outperforming both fixed-threshold Euclidean clustering and DBSCAN in segmentation effectiveness. In areas with significant plant adhesion, although HDBSCAN could distinguish density differences, it still struggled to accurately segment interpenetrating branches. By dividing the total group segmentation time by the number of plants segmented by each algorithm, we calculated the average processing time per plant. Among the four algorithms, the localization and growth method proposed in this study, incorporating plant-level topological constraints and based on a continuous cylindrical model of the main stem, more effectively guided segmentation boundaries and demonstrated significant advantages in handling complex intersecting structures. Meanwhile, due to its superior ability to accurately detect individual plants within the group, it achieved the shortest average processing time per plant.

**Table 7:** Comparison of computational time among different point cloud segmentation methods.

Method	*T*_tol_/s			*T*_avg_/s		
	VARc94	VARf6	VARx36	VARc94	VARf6	VARx36
DBSCAN	131.47	130.48	136.41	1.66	1.61	1.82
Euclidean	281.29	279.17	291.86	3.23	2.94	3.14
HDBSCAN	292.86	293.86	294.86	2.61	2.56	2.50
Ours	174.34	174.67	181.89	1.31	1.32	1.32

### 3.3 Phenotypic trait extraction

**Figure 9:**
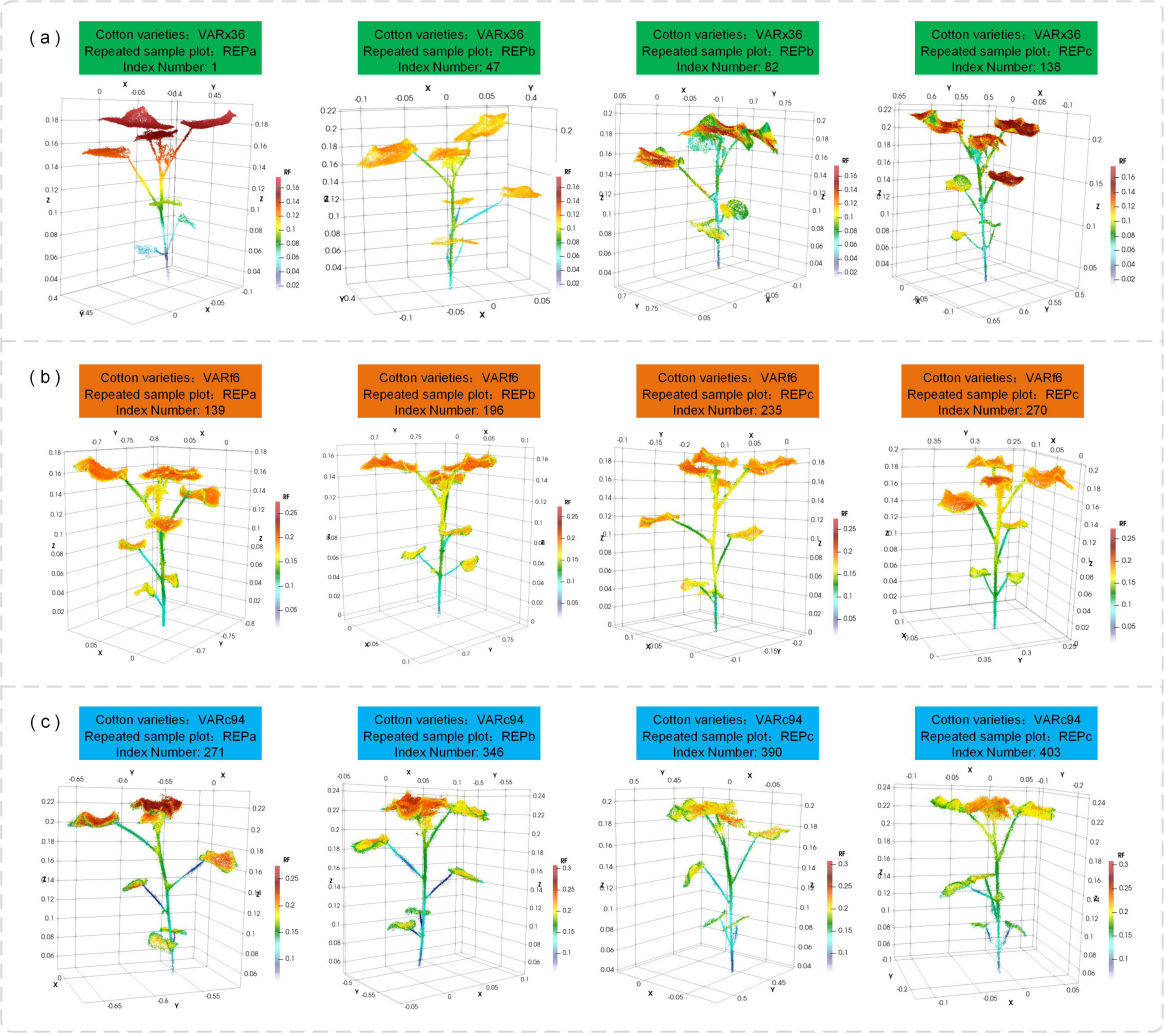
Single plant point clouds after instance segmentation: **(a)** VARx36 variety, plant Index numbers 1-138; **(b)** VARf6 variety, plant Index numbers 139-270; **(c)** VARc94 variety, plant Index numbers 271-403.

**Figure 9** displays partial plant point clouds after instance segmentation. We obtained a total of 403 complete cotton plant point clouds, and a unique information system was established for each plant, primarily storing the cotton variety, replicate number, and Index Number within the entire sample. The analyzed phenotypic information will also be supplemented in the information system of the corresponding plant. This system uses plant localization points as a benchmark for spatial positioning and is effective throughout the entire growth cycle of the cotton.

**Figure 10:**
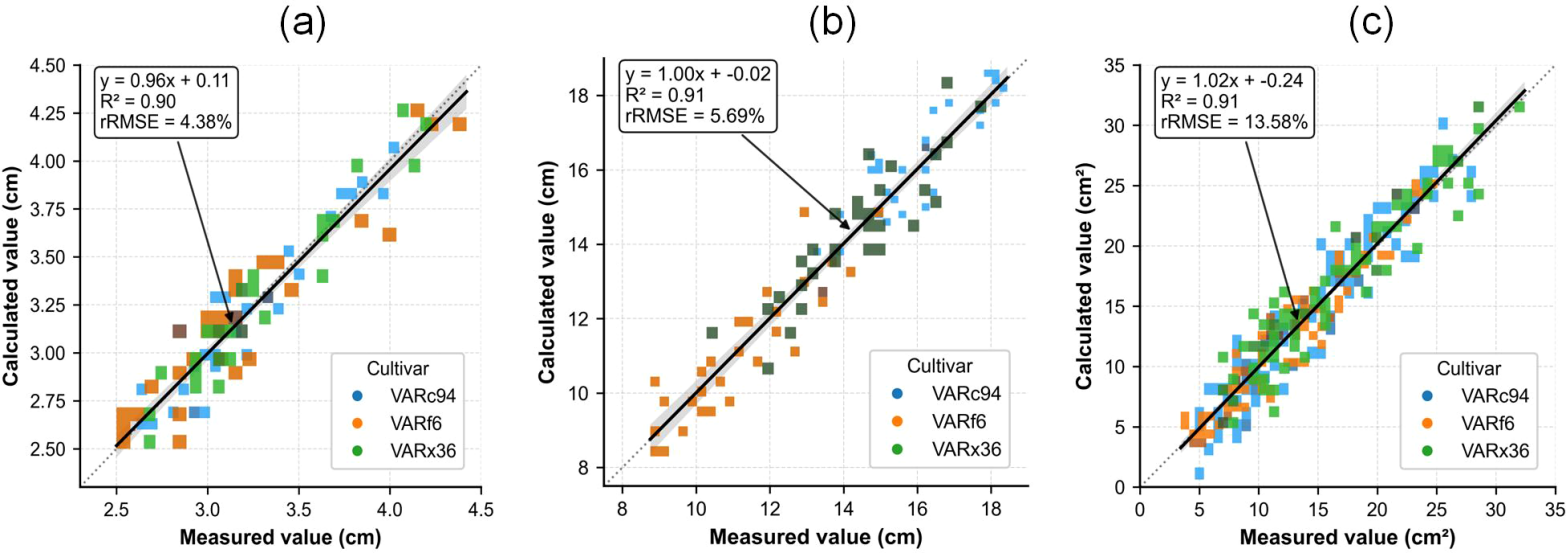
Correlation analysis between predicted and measured cotyledon node height, plant height, and leaf area in cotton. **(a)** Linear regression between predicted and measured cotyledon node height. **(b)** Linear regression between predicted and measured plant height. **(c)** Linear regression between predicted and measured leaf area.

**Figure 10** presents the correlation analysis between the predicted and observed values of cotton cotyledon node height, plant height, and leaf area. The results demonstrate that extracting plant phenotypic traits based on 3D point cloud data achieves high accuracy: the coefficient of determination (*R*^2^) for cotyledon node height prediction is 0.90 (95% CI: 0.87–0.92), with a relative root mean square error (rRMSE) of 4.38% ± 0.51%. The *R*^2^ for plant height prediction is 0.91 (95% CI: 0.89–0.93), with an rRMSE of 5.69% ± 0.63%. For leaf area prediction, the *R*^2^ reaches 0.91 (95% CI: 0.88–0.94), with an rRMSE of 13.58% ± 1.22%. These statistics not only confirm the core accuracy of the method, but their accompanying confidence intervals and variability measures also quantify the stability of the estimates, providing a more robust basis for the reproducibility and comparability of the results.

**Figure 11:**
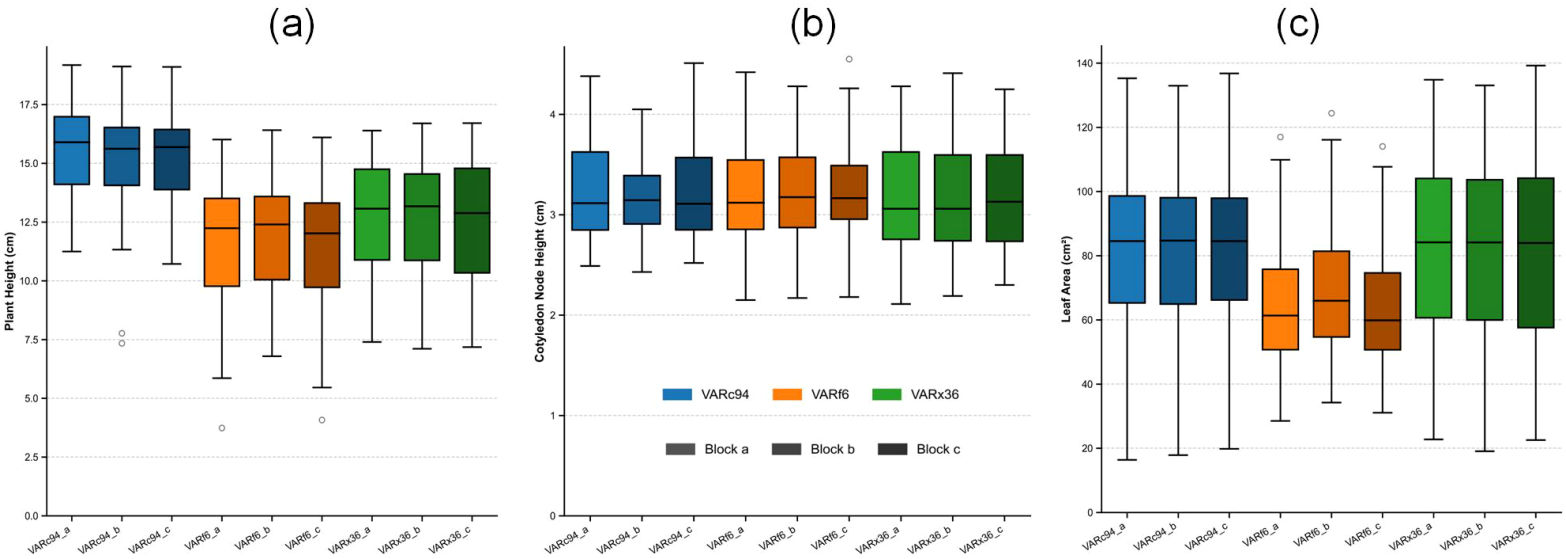
Distribution ranges and error bars of cotyledon node height, plant height, and leaf area traits obtained from cotton plants. **(a)** Distribution range and error bars for cotyledon node height; **(b)** Distribution range and error bars for plant height; **(c)** Distribution range and error bars for leaf area. In the figure, blue denotes the VARc94 cultivar, orange represents VARf6, and green indicates VARx36; varying shades are used to distinguish different sampling plots within each cultivar.

**Figure 11** the distribution ranges and error bars for the acquired traits of cotyledon node height, plant height, and leaf area. The distribution of cotyledon node height was relatively stable across varieties, whereas plant height and leaf area exhibited significant variation in numerical distribution, reflecting the varietal characteristics of cotton. These results indicate substantial differences in growth among cotton varieties. And the PhenotypeAI method proposed in this paper can effectively obtain these differences.

**Figure 12:**
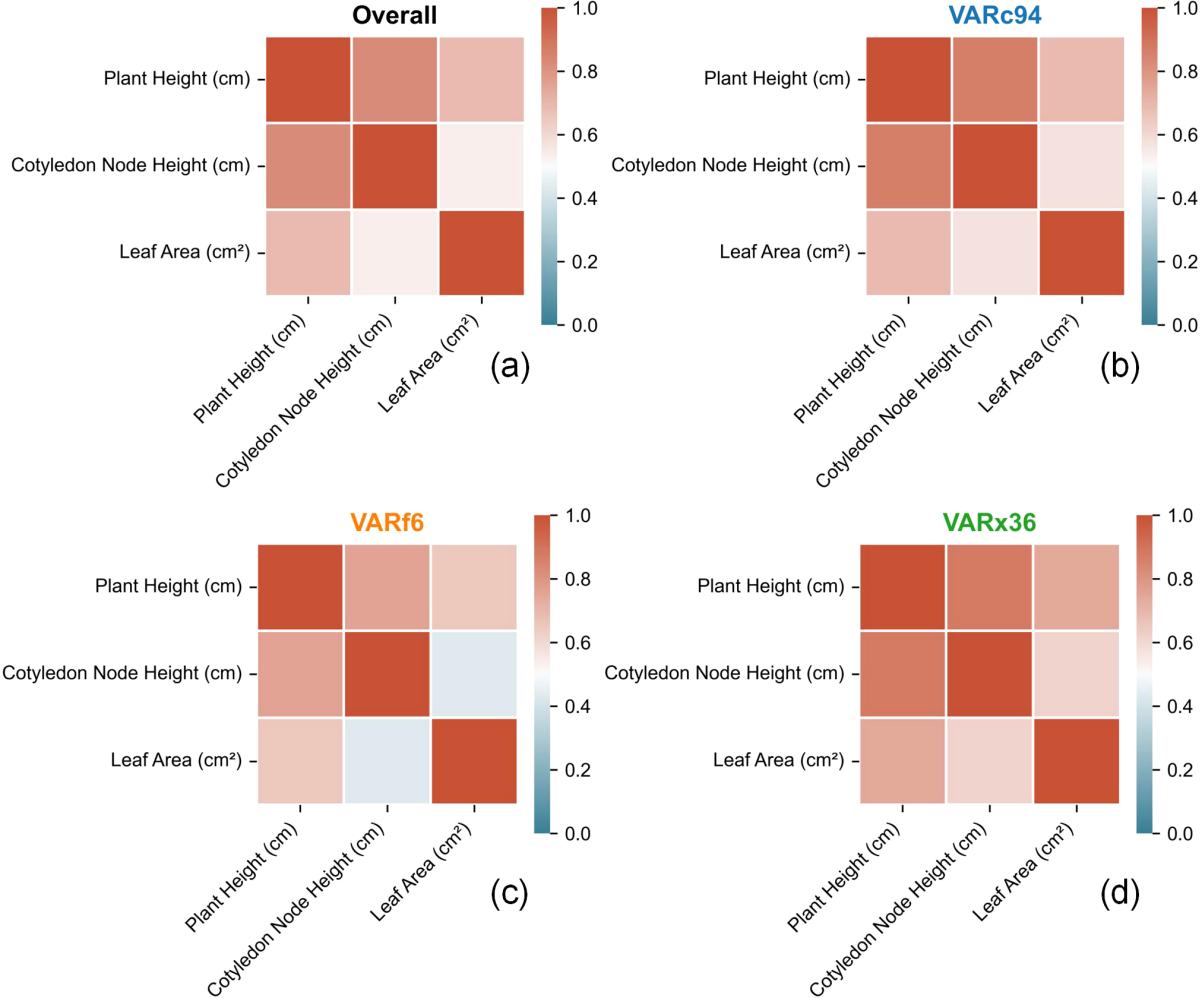
Correlation analysis among cotyledonary node height, plant height, and leaf area traits in different cotton cultivars. **(a)** Correlation analysis among cotyledonary node height, plant height, and leaf area for all cotton plants; **(b)** Correlation analysis among these traits for VARc94 cultivar; **(c)** Correlation analysis for VARf6 cultivar; **(d)** Correlation analysis for VARx36 cultivar.

**Figure 12** Correlation Analysis Among Cotyledon Node Height, Plant Height, and Leaf Area Traits Across Cotton Varieties. The analysis results in the figure below indicate that cotyledon node height and plant height traits exhibit strong correlations among cotton varieties, while the correlation between plant height and leaf area traits is the weakest. The correlation between cotyledon node height and leaf area traits is also the weakest. This aligns with the previously obtained cotyledon node distribution results, primarily because the differences in cotyledon node height traits among cotton varieties at this stage are relatively small, whereas the differences in plant height and leaf area traits are more pronounced. .

**Table 8** Time consumption analysis for cotton trait extraction using PhenotypeAI. The table records the total extraction time for cotyledonary node height, plant height, and leaf area traits across all cultivars and sampling plots, as well as the average extraction time per individual plant for these traits. Overall, the extraction time for cotyledonary node height, plant height, and leaf area in each sampling plot ranged from a minimum of 34.72 seconds to a maximum of 39.79 seconds, while the extraction time per individual plant ranged from 0.85 to 0.93 seconds.

**Table 8:** Time efficiency assessment of cotton phenotypic trait extraction via PhenotypeAI.

Varieties	VARc94			VARf6			VARx36		
	a	b	c	a	b	c	a	b	c
***T***_eta_ **/s**	35.34	38.69	37.65	34.86	34.72	35.93	39.79	37.46	35.61
***T***_avg_ **/s**	0.82	0.86	0.84	0.79	0.81	0.80	0.83	0.81	0.81

## 4 Discussion

### 4.1 PhenotypeAI markedly outperforms sfm-mvs and nerf methods in reconstruction detail

Compared with SFM-MVS and NeRF methods, PhenotypeAI achieved a peak signal-to-noise ratio (PSNR) of 30.0 dB in reconstruction, producing point clouds with the lowest noise level while exhibiting the highest completeness and fineness of plant point clouds. This is crucial for enhancing the success rate of point cloud instance segmentation and the accuracy of phenotypic information extraction. The core advantage of PhenotypeAI lies in its adoption of an explicit discrete representation based on 3D Gaussian Splatting (3DGS). This representation describes a scene as a collection of Gaussian ellipsoids with optimizable parameters, where the scale and orientation of local geometry are directly controlled through covariance matrices. For thin structures such as cotton stems, 3DGS can accurately capture their morphology by arranging slender and directionally strong Gaussian primitives, avoiding the detail blurring caused by the smoothness prior and numerical integration in implicit continuous representations like NeRF. As a result, PhenotypeAI enables more faithful fine-scale geometric reconstruction. In complex cotton canopy scenarios, PhenotypeAI demonstrates significant advantages, requiring only 1.1–1.4 hours to complete point cloud modeling, which is over 90% faster than NeRF-based algorithms, thereby highlighting its potential to advance plant phenotyping analysis.

### 4.2 PLZ for the management of field information and phenotypic traits of individual cotton plants

The accurate localization of plants within a population is crucial for managing phenotypic traits, and a plant localization zone enables the long-term, time-series tracking of individual plant traits. This study leverages the independent growth characteristics of cotton plant stems. By extracting the point cloud of the plant community after ground removal at a specific height layer, root points of individual plants are identified through density-based clustering, and the positioning points for each plant are calculated. This localization point can establish a long-term mapping relationship between the plant’s positional information and its phenotypic data, thereby aiding in the management of plant-level phenotypic information. The proposed method is particularly suitable for high-standard farmlands implementing precision agronomic practices, where interference from robust weeds and other complex factors is minimized. In future work, we plan to incorporate stem diameter, branching angle, and spectral features at specific heights to enhance the generalization capability of the method in complex, non-standard scenarios.

### 4.3 PhenotypeAI has the advantages of high efficiency and low computational cost in population point cloud instance segmentation

Instance segmentation of the population point cloud is an important component for extracting plant traits. In this study, the segmentation accuracy for cotton plant instances reached 88.89% 93.18%, with completion time reduced to just 56.22 seconds, significantly improving efficiency. Additionally, there are several deep learning-based point cloud segmentation methods (Chen et al., 2025). manually labeled cotton point clouds using CloudCompare software and employed the 3D deep neural network PointNet++ for population point cloud segmentation to extract individual cotton plants and the population, achieving a cotton detection rate of R = 86.3%. Although deep learning is currently a very popular method for point cloud segmentation, it relies on large amounts of data for training. Manually labeling this data is error-prone, labor-intensive, and costly. Current deep learning methods also require downsampling the point cloud to a very low resolution, which leads to the loss of many potential geometric features of small organs and plants. The PhenotypeAI method proposed in this study does not require data labeling and training, which saves time and cost, and it exhibits good segmentation performance. Its high efficiency and low computational cost provide technical support for the precise analysis of field phenotypic traits for efficient cotton breeding.

### 4.4 Future work

This study demonstrates that the integration of smartphone-based imaging with Gaussian splatting rendering techniques enables efficient acquisition of point cloud data in cotton fields, effectively accomplishes instance segmentation of cotton populations, and accurately estimates cotyledon node height, plant height, and leaf area of cotton plants. Specifically, the PhenotypeAI methodology achieved a PSNR of 30.0 dB in cotton field reconstruction, with instance segmentation accuracy reaching a minimum of 88.89% and an extraction rate of 100%. In cotton phenotypic trait extraction, plant height exhibited the highest accuracy and lowest error. The cotyledon node height and leaf area traits also satisfied the precision requirements essential for breeding applications. Additionally, we propose an effective smartphone-based method for collecting point cloud data in cotton breeding fields, generating currently scarce point cloud datasets for cotton fields and individual plants. Final experimental results demonstrate that our PhenotypeAI method enables high-throughput, low-cost outdoor cotton reconstruction, instance segmentation, and phenotypic information extraction. This provides technical support for cotton growth monitoring, yield prediction, precision pesticide application, and scientific management. The core idea of this method is unsupervised instance segmentation based on geometric localization of the main stem. For other crops with distinct main stems or branches, such as corn and tomatoes, this workflow is directly transferable. For crops without obvious upright main stems, such as Chinese cabbage and kale, the adaptability accuracy of the current method will significantly decrease. In future research, we will prioritize robust reconstruction in dynamic scenarios and strive to advance the method from standard breeding trial fields to applications in unstructured cotton fields.

## 5 Conclusion

This study successfully developed an innovative method named PhenotypeAI. This technology enables high-precision, low-cost 3D reconstruction of cotton plant populations under field conditions using video footage captured by smartphones. It simultaneously supports point cloud instance segmentation, thereby extracting key phenotypic traits from individual plants. This approach fully demonstrates the immense potential of combining portable smartphone devices with 3D point cloud technology for acquiring cotton phenotypic data in the field. We conducted field trials at a modern cotton breeding base to validate the proposed method. Experimental data demonstrated that point cloud reconstruction was achieved using smartphone-captured canopy images of cotton fields, with a peak signal-to-noise ratio (PSNR) of 30.0 dB and a point cloud density of 394 points per square centimeter. The average accuracy of instance segmentation at the population level reached 91.35%, successfully constructing 3D point cloud models for 403 high-quality cotton plants. The average processing time per plant was 1.3 seconds. Analysis of key plant traits-FCNH (first cotyledon node height), PH (plant height), and LA (leaf area)-exhibited correlation coefficients (*R*^2^) of 0.90, 0.91, and 0.91, respectively, with relative root mean square errors (rRMSE) of 4.38%, 5.69%, and 13.58%. Individual plant analysis completed in as little as 0.79 seconds. Overall, this study provides a low-cost solution for high-throughput field phenotyping of cotton, offering significant value for smart breeding, growth monitoring, and scientific field management. This method holds promise as a technical benchmark for modern breeding and efficient cultivation practices.

## Data Availability

The original contributions presented in the study are included in the article/supplementary material. Further inquiries can be directed to the corresponding authors.
